# Unveiling the glymphatic system’s impact on neurodegenerative diseases: a comprehensive bibliometric analysis (2012–2024)

**DOI:** 10.3389/fnagi.2025.1598608

**Published:** 2025-09-24

**Authors:** Younian Wang, Qiangsheng Liu, Junyi Wu, Kun Meng, Lanting Zhou, Fan Ye

**Affiliations:** ^1^Hubei University of Arts and Science, Xiangyang, China; ^2^Department of Anesthesiology, Xiangyang Central Hospital, Affiliated Hospital of Hubei University of Arts and Science, Xiangyang, China; ^3^Institute of Neuroscience and Brain Science, Xiangyang Central Hospital, Affiliated Hospital of Hubei University of Arts and Science, Xiangyang, China

**Keywords:** glymphatic system, neurodegenerative diseases, Aquaporin-4, Alzheimer’s disease, bibliometrics

## Abstract

**Background:**

The glymphatic system is a crucial factor in the development of neurodegenerative diseases (NDs) and holds substantial promise for therapeutic strategies. However, despite its growing significance, detailed bibliometric investigations specifically targeting the interplay between the glymphatic system and NDs are still sparse. This study pioneers a comprehensive bibliometric analysis to delineate the intellectual framework and emerging trajectories in glymphatic system research associated with NDs.

**Materials and methods:**

We conducted an exhaustive search of the literature on the glymphatic system and NDs using the Web of Science Core Collection (WoSCC), Scopus, and Pubmed Central (PMC) databases. VOSviewer was applied for bibliometric analysis and visualization to scrutinize geographical distribution, institutional distribution, inter-journal connections, and keyword prevalence. Additionally, CiteSpace was utilized to intuitively explore and analyze journal interactions and citation dynamics. The bibliometrix R package was employed to construct visualized networks of international collaborations, as well as the relationships among authors, keywords, and journals. Data visualization was further enhanced with the aid of the WPS Office.

**Results:**

A total of 865 relevant publications were retrieved, covering 92 countries, 1,471 research institutions, and 367 academic journals. Over the past thirteen years, both the number of publications and citations related to the glymphatic system in NDs have shown a significant upward trend, with neuroscience being the primary research field. Chinese authors over the world published the largest number of articles, while China and United States rank the highest in terms of publication and citation counts, also both demonstrate extensive international collaboration. And the *Frontiers series journal* publishes the most papers in this field, while *Brain* is the most frequently cited journal. The University of Rochester is the top-producing institution with an internally well-known Chinese research group. Keyword analysis highlights the glymphatic system, Alzheimer’s disease (AD), cerebrospinal fluid, and aquaporin 4 (AQP4) as core topics, with AQP4 polarization identified as a key regulatory factor for metabolic waste clearance. The main NDs under investigation are AD and Parkinson’s disease (PD).

**Conclusion:**

This study offers a comprehensive preliminary dissection of the research landscape, identifying current focal points and potential future directions in glymphatic system research related to NDs. By leveraging multiple bibliometric approaches, this study provides valuable insights into this burgeoning field.

## 1 Introduction

Neurodegenerative diseases (NDs) manifest through progressive neuronal degeneration in specific CNS regions, encompassing Alzheimer’s disease (AD), Parkinson’s disease (PD), multiple sclerosis (MS), amyotrophic lateral sclerosis (ALS), and Huntington’s disease (HD) (Guo et al., 2022). These conditions share core neuropathological signatures including chronic neuroinflammation and pathological protein conglomerates. AD is pathologically defined by two hallmark features: extracellular deposition of *β*-amyloid (Aβ) plaques and intraneuronal accumulation of hyperphosphorylated Tau protein-laden neurofibrillary tangles, a dual pathogenic mechanism extensively corroborated in modern neuropathological paradigms (Andrade-Guerrero et al., 2023). PD presents selective dopaminergic neuron loss within the substantia nigra pars compacta accompanied by *α*-synuclein-enriched Lewy bodies (Zhou et al., 2024). HD stems from abnormal Cytosine-Adenine-Guanine (CAG) trinucleotide expansion in the huntingtin (HTT) gene, driving progressive striatal and cortical neuron degeneration (Chang et al., 2024; Thipani Madhu et al., 2024). These pathogenic aggregates not only disrupt neuronal equilibrium but also amplify neural injury through innate immune activation and oxidative cascades (Kritsilis et al., 2018). Of critical concern is the global surge in NDs prevalence due to population aging, which imposes an escalating socioeconomic strain on healthcare infrastructures (Bhattacharya et al., 2022). NDs trigger irreversible neurological impairments including cognitive deterioration, motor deficits, and emotional dysregulation, which collectively degrade patients’ functional independence. This clinical reality underscores the imperative to decode disease mechanisms and formulate precision interventions. The brain’s glymphatic network first mapped in 2012 through pioneering rodent studies serves as a macroscopic clearance pathway mediating cerebrospinal-interstitial fluid exchange (Iliff et al., 2012). This pathway facilitates efficient clearance of neurotoxic metabolites like Aβ and Tau proteins through arteriole pulsation-driven paravascular flux, a process modulated by astrocytic endfoot aquaporin 4 (AQP4) water channel polarization (Naganawa et al., 2024). Notably, glymphatic activity exhibits circadian rhythmicity, with enhanced function during non-rapid eye movement sleep, suggesting that sleep disturbances may contribute to NDs progression via impaired metabolic clearance (Gottesman et al., 2024). Accumulating evidence indicates that glymphatic dysfunction disrupts the homeostasis of pathogenic proteins (e.g., Aβ, *α*-synuclein), directly implicating this system in the etiopathogenesis of AD, PD, and other NDs (Beschorner and Nedergaard, 2024; Yamada and Iwatsubo, 2024). Thus, targeting glymphatic pathways emerges as a promising therapeutic strategy for NDs intervention. With the deepening understanding of the pathophysiological mechanisms of the glymphatic system, researchers are actively developing *in vivo* detection technologies to quantitatively assess its function. In recent years, advanced neuroimaging techniques such as dynamic contrast-enhanced MRI (DCE-MRI), diffusion tensor imaging along the perivascular space (DTI-ALPS), and ultra-high-resolution PET tracers have been employed to quantitatively evaluate human glymphatic transport efficiency in vivo. Naganawa et al. (2024) and Ringstad et al. (2018) successively used intrathecal gadolinium-enhanced DCE-MRI to map cerebrospinal fluid-interstitial fluid (CSF-ISF) exchange in real time at a whole-brain scale, establishing standardized rate and clearance metrics. Zhou et al. (2024) and Premi et al. (2024) further applied DTI-ALPS and multimodal MRI to Parkinson’s disease and genetic frontotemporal dementia cohorts, respectively, demonstrating that reduced glymphatic transport efficiency significantly correlates with motor milestone risk and disease severity. Wilson et al. (2020) and Bah et al. (2023) proposed that novel PET tracers combined with computational fluid dynamics models could elucidate fluid dynamic alterations associated with aging and dementia at the molecular level, attributing glymphatic clearance impairment to diminished CSF pulsatility and reduced vascular compliance. These studies not only provide directly measurable glymphatic functional parameters but also establish a dual foundation—at both imaging and molecular levels—for clarifying the system’s mechanistic role in various NDs.

Bibliometrics, an interdisciplinary field employing mathematical and statistical methodologies to analyze academic literature (Liu et al., 2024b; Sun et al., 2023), provides systematic insights into knowledge structures, collaborative networks, research frontiers, and evolutionary trends within specific domains. Despite significant advancements in glymphatic research related to NDs, a comprehensive bibliometric analysis mapping the intellectual landscape of this field remains absent. This study pioneers the integration of CiteSpace (v6.3. R1), VOSviewer (v1.6.20), and Bibliometrix R package to conduct a multidimensional bibliometric analysis of original research articles on the glymphatic system in NDs published between 2012 and 2024. By constructing scientometric knowledge maps, we aim to delineate core research clusters, interdisciplinary intersections, and paradigm shifts, providing strategic guidance for future mechanistic explorations and translational applications in NDs research.

## 2 Materials and methods

### 2.1 Data collection

Selecting multiple academic databases and formulating an effective retrieval strategy are crucial for acquiring high-quality datasets. In this study, the WOSCC (Clarivate 2025-03-01), Scopus (Elsevier 2025-03-01), PMC (NLM 2025-03-01) were selected as the data sources to provide comprehensive support for the literature analysis. WOSCC, which encompasses the Science Citation Index Expanded (SCIE), Social Sciences Citation Index (SSCI), Arts & Humanities Citation Index (A&HCI), and Emerging Sources Citation Index (ESCI), offers authoritative publications in the research field of the glymphatic system and neurodegenerative diseases. Scopus, recognized as the largest abstract and citation database covering a broad range of disciplines, provides extensive peer-reviewed literature. PMC, a full-text repository, offers free and open access to a growing archive of biomedical and life sciences journal literature indexed in PubMed. Given their extensive coverage and high-quality content, WOSCC, Scopus, and PMC were chosen as the primary data sources for this research.

**Figure 1:**
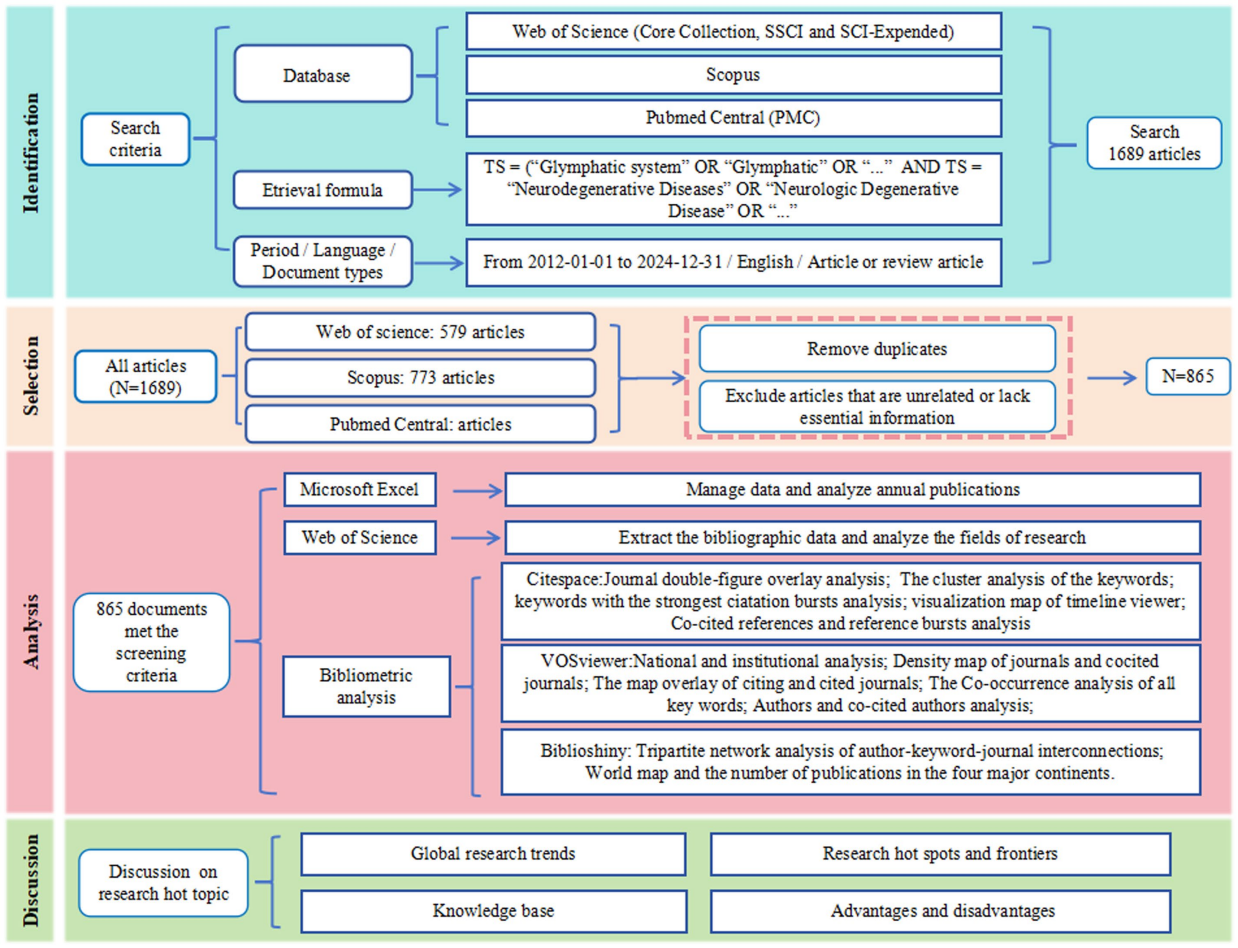
Study flow diagram.

To systematically obtain articles in this field, the search strategy employed the following Boolean query: TS = (“glymphatic system” OR “glymphatic pathway” OR “Glymphatic Pathways” OR “Pathway, Glymphatic” OR “Glymphatic Clearance System” OR “glymphatic” OR “glial lymphatic” OR “Brain Perivascular Spaces” OR “Brain Perivascular Space” OR “Perivascular Space, Brain” OR “paravascular pathway”) AND TS = (“Neurodegenerative Diseases” OR “Degenerative Diseases, Neurologic” OR“Neurologic Degenerative Disease” OR “Degenerative Neurologic Diseases” OR “Degenerative Neurologic Disease” OR “Neurologic Disease, Degenerative” OR “Neurologic Diseases, Degenerative” OR “Nervous System Degenerative Diseases” OR “Neurodegenerative Disorders” OR “Neurodegenerative Disorder” OR “Neurologic Degenerative Conditions” OR “Degenerative Condition, Neurologic” OR “Degenerative Conditions, Neurologic” OR “Neurologic Degenerative Condition” OR “Neurologic Degenerative Diseases” OR “Degenerative Diseases, Nervous System” OR “Degenerative Neurologic Disorders” OR “Degenerative Neurologic Disorder” OR “Neurologic Disorder, Degenerative” OR “Neurologic Disorders, Degenerative” OR “Degenerative Diseases, Spinal Cord” OR “Degenerative Diseases, Central Nervous System”OR “Alzheimer’s Disease” OR “Parkinson’s Disease” OR “Multiple Sclerosis” OR “Amyotrophic Lateral Sclerosis” OR “Huntington’s Disease”). The document types were specifically designated as “article” and “review article” to ensure the high quality of the data sources. Review articles were selected for their capacity to synthesize high-quality research findings, identify research gaps, and propose potential solutions. The publication date was restricted to the period from January 1, 2012, to December 31, 2024, and the language was limited to English. Initially, a total of 1,689 articles were retrieved, with 579 from the WOSCC, 773 from Scopus, and 337 from PMC. After removing duplicates and excluding articles that were unrelated to the theme or lacked essential information such as DOI, volume, start page, keywords, and references, a final dataset of 865 publications was retained for analysis (Figure 1).

### 2.2 Data analysis

For computational bibliometric evaluation, we employed three validated analytical frameworks: VOSviewer (v1.6.20), CiteSpace (v6.3. R1), and Bibliometrix R package (v4.1.3). The VOSviewer platform was configured to perform geospatial publication distribution profiling across geopolitical entities, institutional affiliations, and journal platforms, concurrently analyzing co-citation networks and term frequency matrices through predefined operational parameters: full-text enumeration methodology, multi-tiered weighting schema (document-based metrics for geopolitical/institutional/journal entities, citation-weighted indices for co-cited periodicals, frequency-adjusted measures for author-designated terminologies), association strength normalization protocols, and clustering criteria (resolution index = 1.00, minimum node threshold = 1, edge weight exclusio*n* = 0). The Java-architected CiteSpace environment facilitated temporal network decomposition through chronographic segmentation (2012–2024 with annual intervalization), g-index scaled network topology (*k* = 14 coefficient), reference co-citation node typology, per-slice top-50 entry filtration, and Log-Likelihood Ratio clustering algorithm implementation, enabling dynamic visualization of journal-reference network architectures. The Bibliometrix R package, operating within RStudio 2023.03.1 (R v4.3.2 computational backend), executed international collaboration cartography and tripartite relationship visualization (author-keyword-journal nexus) alongside emergent term detection protocols constrained by temporal demarcation (2012–2024 baseline), lexical inclusion thresholds (minimum frequency = 5, annual term quota = 3). Supplementary bibliometric indicators encompassing publication volumetrics, authorship distribution patterns, and citation trajectory analyses were processed through WPS Office’s advanced analytical suite for graphical representation.

## 3 Results

### 3.1 Fundamental quantitative data

865 articles written by 4,544 authors from 1,471 institutions in 92 countries were identified using the specified search strategy. Totaling 50,100 references from 7,138 different articles, these papers were published in 367 journals.

### 3.2 Quantitative analysis of publications

**Figure 2:**
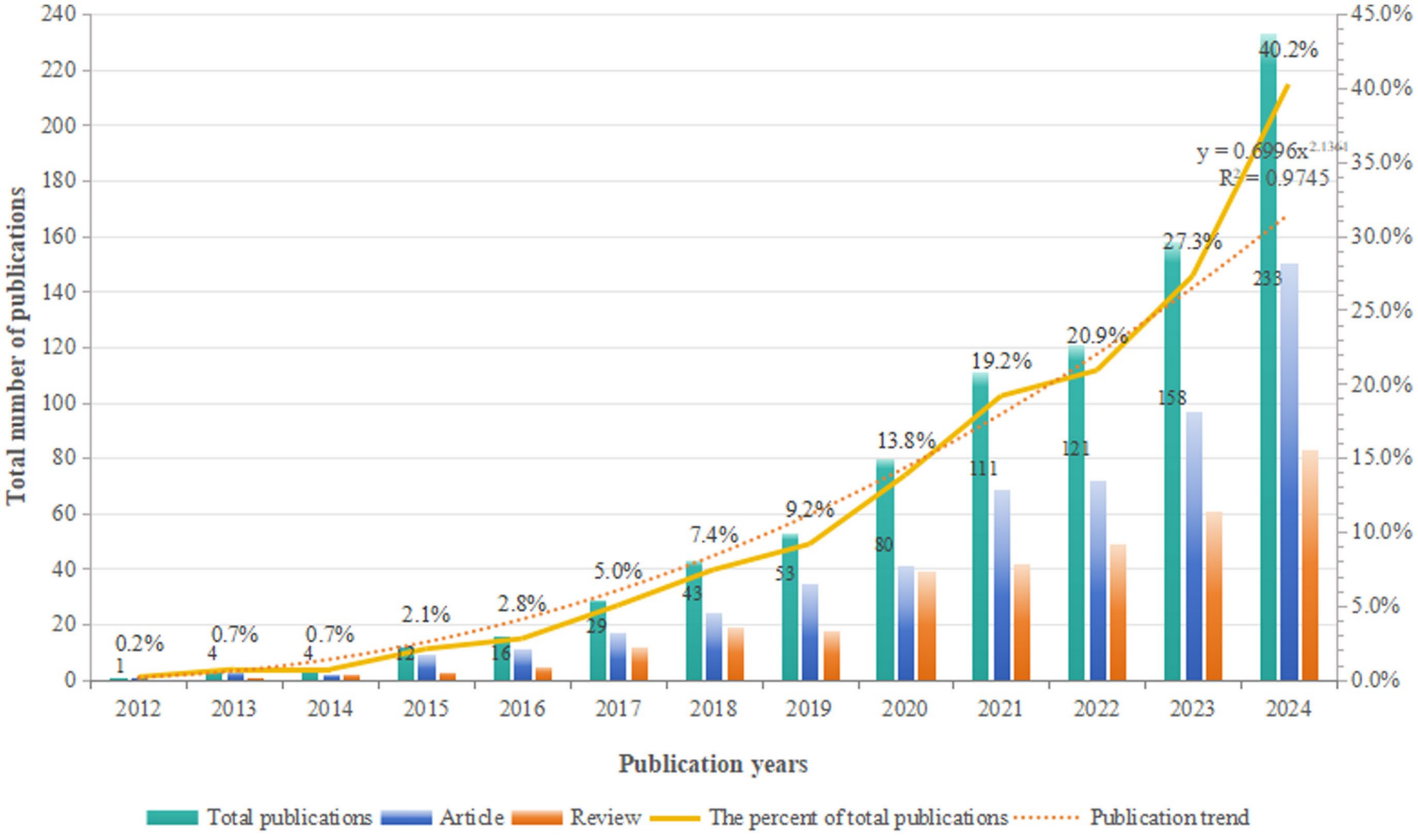
Annual output of research on glymphatic system in NDs from 2012 to 2024.

To reveal the development trends of glymphatic system research in the field of NDs, we integrated literature resources from three authoritative databases: Web of Science, Scopus, and PubMed Central. After a rigorous process of deduplication and screening, we included 865 high-quality articles for in-depth analysis. Through systematic categorization of publication types, our analysis revealed that original articles (*n* = 531, 61.39%) predominated over review articles (*n* = 334, 38.61%), with an approximate ratio of 1.59:1. This distribution underscores the field’s sustained emphasis on empirical investigation. From a macro-development perspective, as shown in Figure 2, the number of publications in this field has shown a significant upward trend, indicating a continuous increase in research activity. Based on the changes in annual publication volume, the research development from 2012 to 2024 can be divided into four phases: the initial phase (2012–2016), the early development phase (2017–2019), the rapid growth phase (2020–2023), and the current phase (2024 to present). During the initial phase, although the publication volume was low, these studies laid a crucial foundation for the in-depth exploration of the role of the glymphatic system in NDs. In the early development phase, there was a significant increase in publication volume compared to the initial phase, with an average annual output of approximately 125 articles. The rapid growth phase was characterized by a sharp increase in publication volume, with the number exceeding 150 articles in 2023. This marked acceleration reflects the growing attention from the academic community to the role of the glymphatic system in NDs. Notably, in 2024, the publication volume reached 233 articles, accounting for 40.2% of the total publications, and the growth trend shows no signs of slowing down. This trend indicates the increasing importance of the glymphatic system in NDs research, and it is anticipated that more relevant findings will be published in the future.

### 3.3 Distribution of research fields

**Figure 3:**
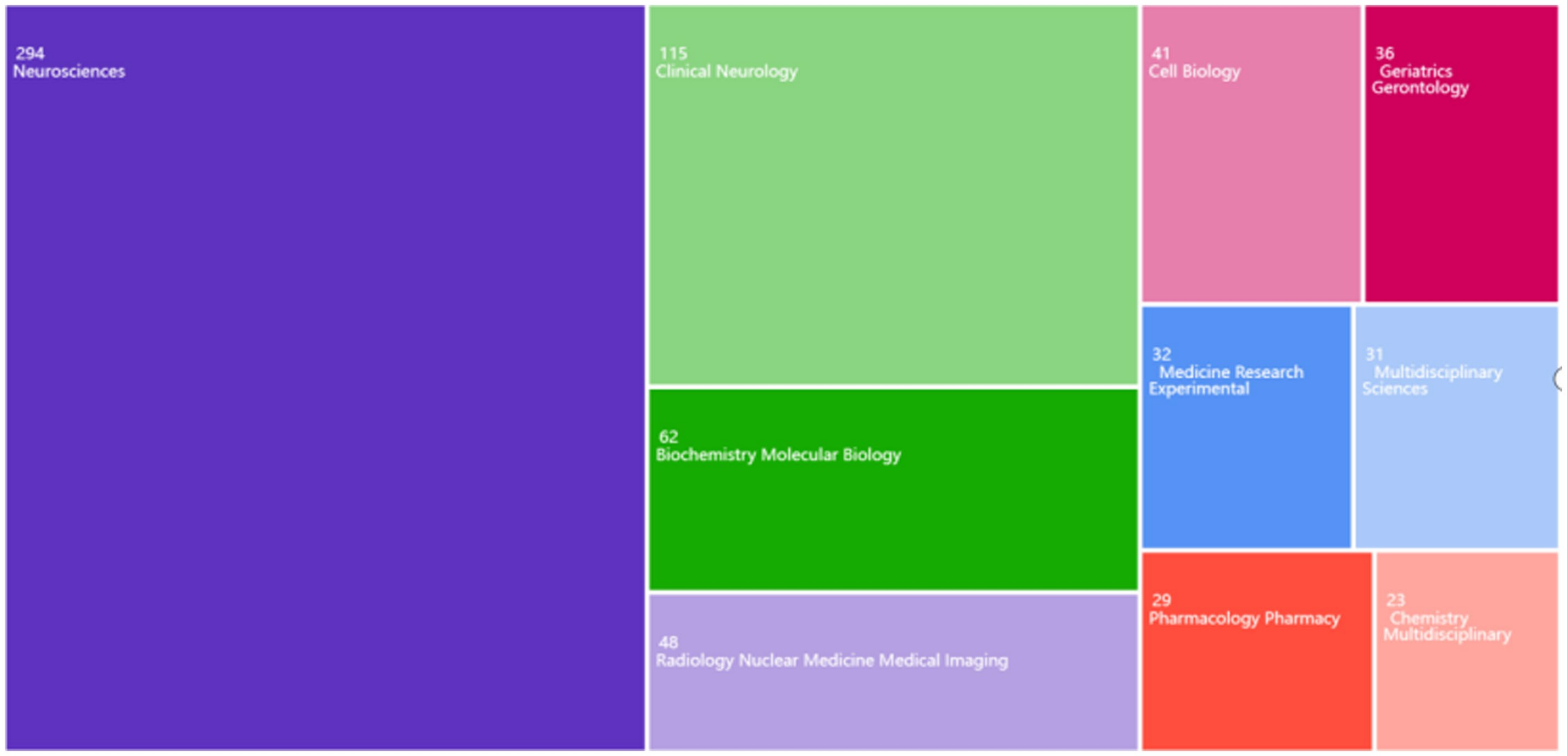
Distribution of major research fields in the study of the glymphatic system in NDs from 2012 to 2024.

The Web of Science database was utilized to analyze various research fields and identify the top 10 fields (Figure 3). In the study of the glymphatic system in NDs, Neuroscience (294 articles, accounting for 50.78%), Clinical Neurology (115 articles, 19.86%), and Biochemistry Molecular Biology (62 articles, 10.71%) are the primary research areas. Notably, Radiology Nuclear Medicine Medical Imaging (48 articles, 8.29%) also occupies a significant proportion. This indicates that research on the glymphatic system in NDs focuses not only on basic research and clinical translation but also on imaging assessment.

### 3.4 National and institutional analysis

**Figure 4:**
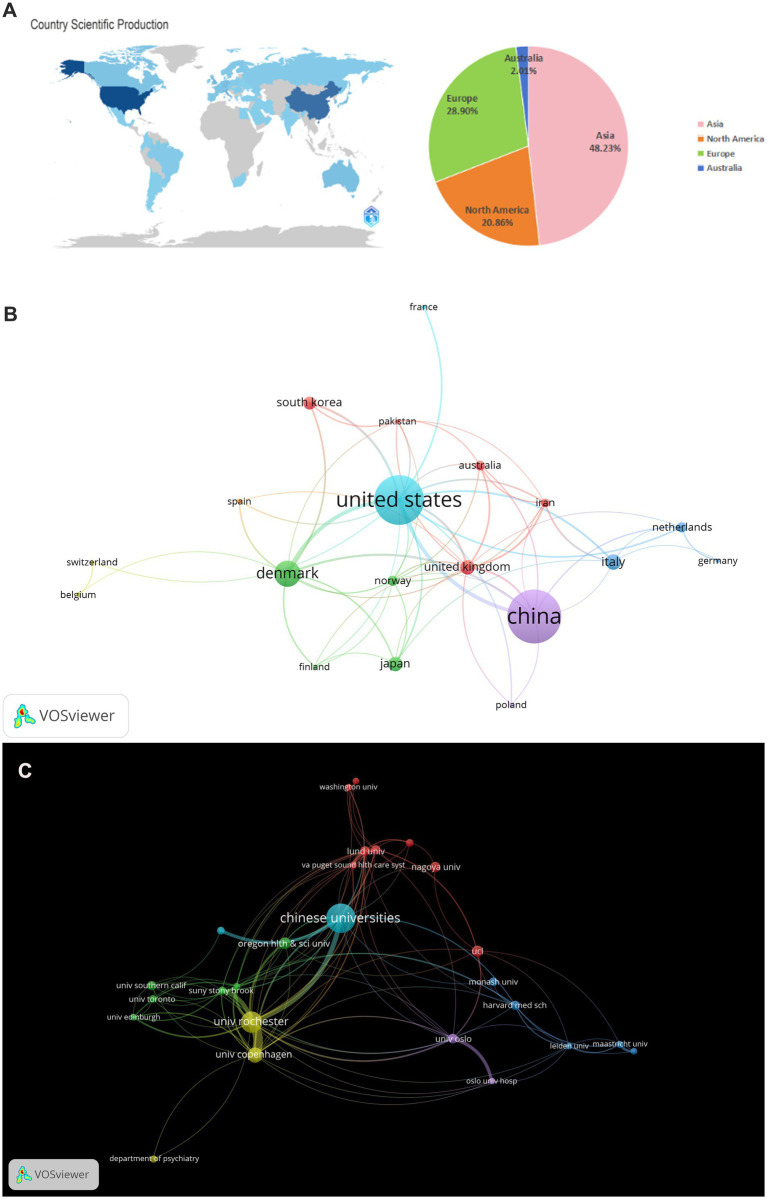
**(A)** World map and the number of publications in the four major continents; **(B)** National visualization analysis of glymphatic system in NDs; **(C)** Institutional visualization analysis of glymphatic system in NDs.

In the field of glymphatic system research in NDs, a total of 865 articles were published by 1,471 different institutions across 92 countries. Based on the VOSviewer analysis platform, this study conducted a systematic quantitative analysis and visualized the research output in this field using the country of the first author as the statistical reference. The results show that the countries of the contributing authors span Asia, Europe, North America, and Australia (Table 1; Figure 4A). From a global regional perspective, Asia (504 articles, 48.23%), Europe (302 articles, 28.90%), and North America (218 articles, 20.86%) have emerged as the three primary contributors to academic output in this field, a distribution pattern that closely aligns with the global landscape of scientific and economic development. At the national level, China (351 articles, 33.59%, 23,411 citations) and the United States (215 articles, 20.57%, 30,531 citations) lead the way in both publication numbers and citation impact, and both countries exhibit extensive international collaboration. Following them are Italy (80 articles, 7.66%, 1,762 citations), Japan (68 articles, 6.51%, 3,723 citations), and Denmark (54 articles, 5.17%, 13,862 citations) (Table 1). These five countries collectively serve as the cornerstone supporting foundational research on the glymphatic system in NDs. To enhance analytical precision, we established a publication threshold of ≥5 per country and consolidated synonymous entries, generating a collaborative network visualization comprising 19 countries to delineate both research productivity and inter-country partnership patterns (Figure 4B). Within this analytical framework, the size of each circle is proportional to a country’s publication output in the target research field; larger circles indicate higher publication volumes. The connecting lines represent collaboration intensity, with thicker lines denoting higher cooperation frequency. For instance, China, which has the largest publication volume, maintains strong research collaborations with the United States, the United Kingdom, Australia, and Netherlands. Meanwhile, the United States primarily engages in extensive cooperation with the China, Denmark, the United Kingdom, and South Korea. It is noteworthy that the countries participating in the international collaboration network are primarily developed countries (Table 1). In addition, we have also compiled statistics on the top 20 institutions by publication volume, with Chinese universities consolidated into a single group (Table 2). Overall, the group of Chinese universities has published the largest number of articles (109 articles, 30.70%). At the level of individual institutions, the University of Rochester in the United States ranks first with 38 published articles (accounting for 10.70%) and an impressive citation count of 11,989. This is followed by the University of Copenhagen in Denmark (22 articles, 6.20%) and Oregon Health & Science University in the United States (17 articles, 4.79%). To further refine our investigation, we applied a publication threshold of ≥6 articles and selected 33 institutions from the initial 1,471 candidates. The data have been incorporated into a network visualization to illustrate the productivity and collaborative connections among institutions (Figure 4C). In this diagram, the diameter of the circles is proportional to the output of each institution, with larger circles indicating greater output. It is evident that the Chinese university group makes the largest overall contribution, while among individual institutions, the University of Rochester has the highest output and citation frequency. The lines connecting circles are weighted according to the frequency of collaboration between institutions—thicker lines indicate closer and more frequent collaboration. For example, particularly close partnerships exist between the Chinese university group, the University of Rochester, and Oregon Health & Science University. The University of Rochester and the University of Copenhagen also maintain strong cooperative ties. Notably, some institutions such as Massachusetts General Hospital and Eindhoven University of Technology have each published five or more articles, but have yet to establish significant collaborations with the core institutions in the field of glymphatic system and NDs research. With the increasing diversity of international collaborations and the rise of cross-border cooperation, communication and collaborative efforts among research institutions are expected to further intensify in the future.

**Table 1:** Country ranking by publication frequency.

Continents	Country	Counts	Citations	Total link strength
Asia (504,48.23%)	China	351 (33.59%)	23,411	26,309
	Japan	68 (6.51%)	3,723	4,767
	South Korea	49 (4.69%)	1852	2,343
	Iran	20 (1.91%)	505	1,439
	Pakistan	6 (0.57%)	97	420
	Indonesia	4 (0.38%)	155	246
	Turkey	3 (0.29%)	6	155
	India	3 (0.29%)	71	148
Europe (302,28.90%)	Italy	80 (7.66%)	1762	4,888
	Denmark	54 (5.17%)	13,862	6,021
	Norway	32 (3.06%)	4,756	2,781
	Netherlands	32 (3.06%)	376	1,455
	United Kingdom	29 (2.78%)	1847	2,543
	Finland	12 (1.15%)	1,274	831
	Poland	9 (0.86%)	118	914
	Sweden	7 (0.67%)	40	714
	Spain	7 (0.67%)	58	254
	Belgium	7 (0.67%)	0	143
	Switzerland	6 (0.57%)	0	146
	Germany	6 (0.57%)	33	71
	France	5 (0.48%)	58	122
	Greece	4 (0.38%)	95	274
	Russia	3 (0.29%)	41	41
	Ireland	3 (0.29%)	94	180
	Hungary	3 (0.29%)	109	116
	Austria	3 (0.29%)	0	36
North America (218,20.86%)	United States	215 (20.57%)	30,531	18,783
	Canada	3 (0.29%)	706	361
Australia (21,2.01%)	Australia	21 (2.01%)	994	1,576

**Table 2:** Ranking of top 20 organizations based on publication frequency.

Rank	Organization	Country	Counts	Citations	Total link strength
1	Chinese Universities	China	109 (30.70%)	8,268	66
2	University of Rochester	United States	38 (10.70%)	11,989	73
3	University of Copenhagen	Denmark	22 (6.20%)	3,693	45
4	Oregon Health and Science University	United States	17 (4.79%)	3,207	14
5	University College London	United Kingdom	16 (4.51%)	607	14
6	Nagoya University	Japan	13 (3.66%)	1,063	7
7	Yale School of Medicine	United States	13 (3.66%)	619	9
8	University of Oslo	Norway	12 (3.38%)	3,847	24
9	Lund University	Sweden	12 (3.38%)	930	20
10	Harvard Medical School	United States	11 (3.10%)	552	20
11	University of Southern California	United States	11 (3.10%)	716	9
12	University of Washington	United States	11 (3.10%)	625	26
13	Monash University	Australia	10 (2.82%)	367	7
14	University of Copenhagen	Denmark	10 (2.82%)	561	11
15	University of Rochester Medical Center	United States	10 (2.82%)	197	4
16	State University of New York at Stony Brook	United States	9 (2.54%)	5,135	30
17	University of Toronto	Canada	8 (2.25%)	797	7
18	Johns Hopkins University	United States	8 (2.25%)	111	7
19	University of Kentucky	United States	8 (2.25%)	753	8
20	Eindhoven University of Technology	Netherlands	7 (1.97%)	1,589	26

### 3.5 Journals and co-cited journals analysis

**Figure 5:**
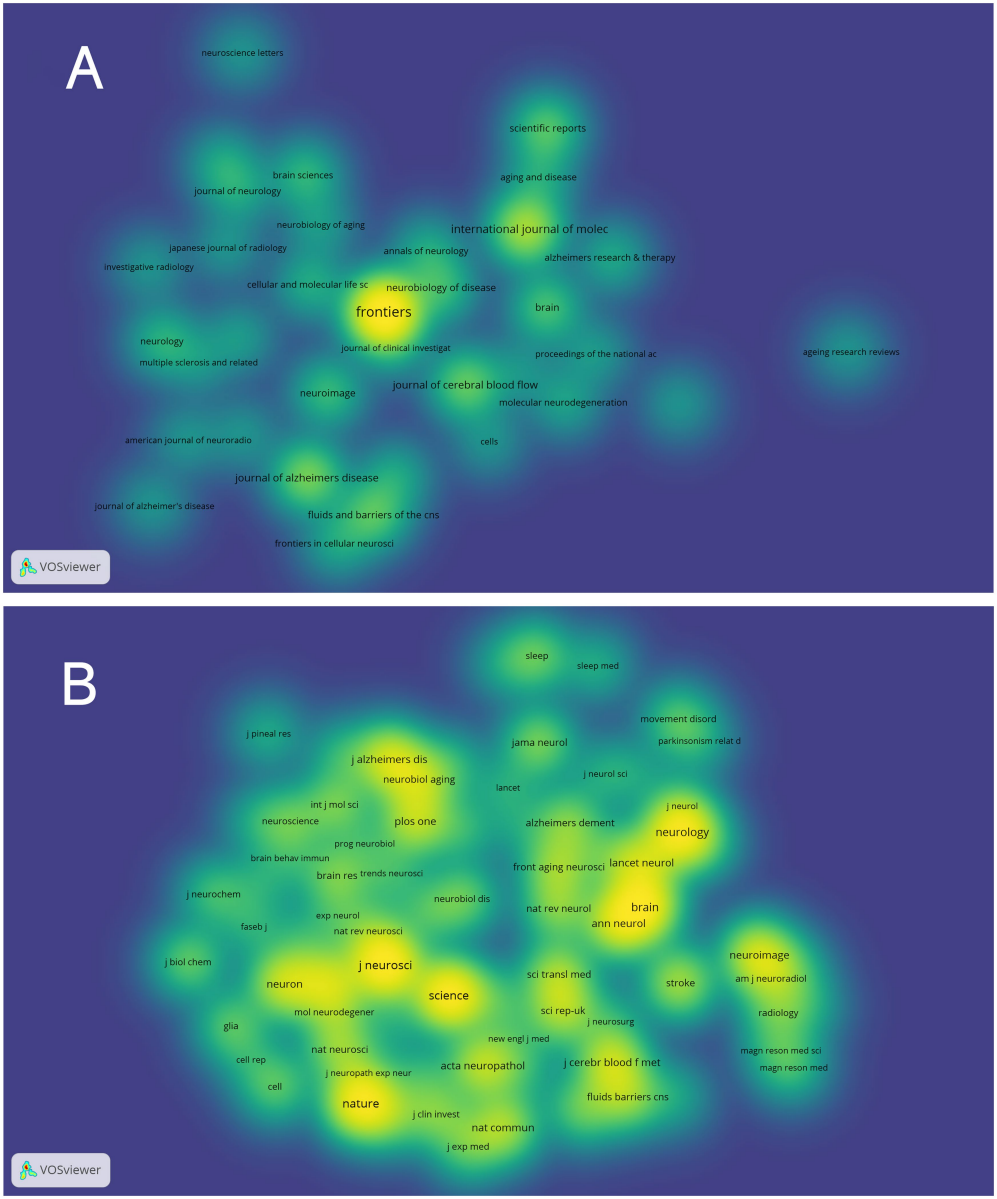
Density map of journals associated with glymphatic system research in NDs; **(A)** Depicts periodicals meeting a threshold of ≥5 published articles (35 journals collectively); **(B)** Includes co-cited journals with ≥150 citations (79 journals aggregately).

**Figure 6:**
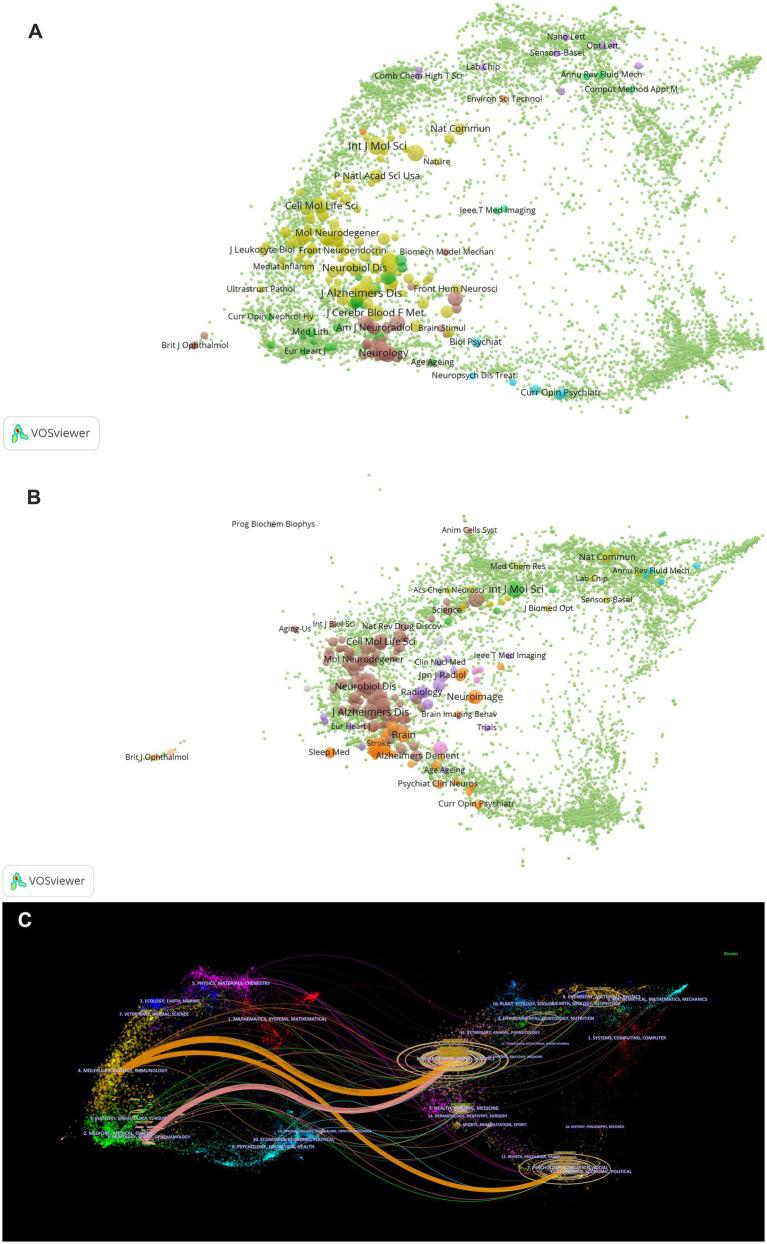
**(A)** The map overlay of citing journals regarding the glymphatic system in NDs; **(B)** The map overlay of cited journals regarding the glymphatic system in NDs; **(C)** Journal double-figure overlay of glymphatic system in NDs.

VOSviewer was employed to analyze journals and co-cited journals (Table 3). The analysis revealed that a total of 865 articles were published across 367 distinct academic journals. This paper presents the top 10 journals ranked by publication count. After consolidating the Frontiers series of journals, they merged them into a more cohesive and strategically significant core category—*Frontiers.* Within this new category, Frontiers tops the overall ranking in terms of publication volume, with 68 articles accounting for 31.3%. Among these, *Frontiers in Aging Neuroscience* (22 articles, 10.1%) and *Frontiers in Neuroscience* (21 articles, 9.7%) have played pivotal roles, emerging as the primary drivers of this success. They are followed by the *International Journal of Molecular Sciences* (29 articles, 13.4%), *the Journal of Alzheimer’s Disease* (21 articles, 9.7%), and *the Journal of Cerebral Blood Flow and Metabolism* (18 articles, 8.3%). The Journal Citation Reports (JCR), developed by Thomson Reuters, is an important metric for assessing the quality of academic journals. JCR categorizes journals into 176 different subject categories and ranks them into Q1, Q2, Q3, and Q4 based on their impact factors (Liu et al., 2024a). Among the top 10 journals, all but *Frontiers in Pharmacology* and *Frontiers in Immunology* published more than 10 papers. Moreover, the majority of these journals are listed in the Q1 section of JCR. Hirsch developed the h-index to assess a journal’s impact on citations and productivity (Hirsch, 2005). In order to reflect the journal’s importance in the area, the h-index calculates the number of papers (h) that have been referenced at least h times (Hou et al., 2022). According to Table 2, *Neuroimage* has the greatest h-index (H-index: 320), followed by Brain (H-index: 308). Among these journals, *Frontiers* leads in total citations (1,112 times), followed by *Brain* (986 times) and the *Journal of Cerebral Blood Flow and Metabolism* (749 times). Additionally, *Brain* has the highest impact factor (IF = 11.9) among these journals. Among the top 10 co-cited journals, *the Journal of Neuroscience* was cited 1,601 times, ranking first, followed by *Science* (1,575 times) and *Nature* (1,367 times). Co-cited journals refer to journals that are cited together in the reference lists of two or more academic publications. If two journals are frequently cited together in the same set of papers, a co-citation relationship exists between them. Analyzing co-cited journals can reveal academic connections between journals, the developmental trajectory of research fields, and the intellectual structure of disciplines. Nine out of the top 10 co-cited journals belong to the Q1 JCR division, with Nature having the highest IF (IF = 50.5). A density map can visually display journals and co-cited journals (Figure 5). Journal overlay maps can visually show the distribution of journals across disciplines; the first map is an overlay of citing journals (Figure 6A), and the second is an overlay of cited journals (Figure 6B). We found that the dual map overlay of journals can visually display the distribution of journals across disciplines and the evolution of citation trajectories. The distribution of journals and the connection between citing and cited journals are accurately depicted by the dual map coverage of journals in Figure 6C. Citation associations are indicated by the colored paths. Citing journals are displayed on the left map, and cited journals are displayed on the right map. Three primary reference paths are shown (Figure 6C). According to the current study, articles from the magazine “Molecular/Biology/Genetics” were frequently referenced by “Molecular/Biology/Immunology” and “Neurology/Sports/Ophthalmology.” Additionally, “Molecular/Biology/Immunology” frequently cited articles from the magazine “Psychology/Education/Social.”

**Table 3:** Top 10 most productive journals and co-cited journals: comparative analysis of publication output and citation impact.

Rank	Journals	Counts	Citations	JCR (IF) (2023)	H-index	Rank	Co-cited journals	Citations	JCR (IF) (2023)	H-index
1	Frontiers series (total)	68 (31.3%)	/	/	/	1	Journal of neuroscience	1,601	Q1 (4.4)	422
	frontiers in aging neuroscience	22 (10.1%)	369	Q2 (4.1)	120					
	frontiers in neuroscience	21 (9.7%)	339	Q2 (3.2)	153					
	frontiers in neurology	14 (6.5%)	139	Q3 (2.7)	105					
	frontiers in pharmacology	6 (2.8%)	247	Q1 (4.4)	62					
	frontiers in immunology	5 (2.3%)	18	Q1 (5.7)	155					
2	International journal of molecular sciences	29 (13.4%)	432	Q1 (4.9)	114	2	Science	1,575	Q1 (44.8)	1,058
3	Journal of Alzheimers disease	21 (9.7%)	447	Q2 (3.4)	115	3	Nature	1,367	Q1 (50.5)	1,096
4	Journal of cerebral blood flow and metabolism	18 (8.3%)	749	Q1 (4.9)	177	4	Neurology	1,316	Q1 (8.4)	331
5	Scientific reports	16 (7.4%)	375	Q1 (3.8)	149	5	Brain	1,315	Q1 (11.9)	308
6	Fluids and barriers of the cns	16 (7.4%)	120	Q1 (5.9)	65	6	Journal of Alzheimers disease	823	Q2 (3.4)	115
7	Brain	14 (6.5%)	986	Q1 (11.9)	308	7	Neuron	930	Q1 (16.2)	530
8	Neurobiology of disease	12 (5.5%)	587	Q1 (5.1)	151	8	Neuroimage	924	Q1 (4.7)	320
9	Neuroimage	12 (5.5%)	268	Q1 (4.7)	320	9	Annals of neurology	907	Q1 (8.1)	273
10	Brain sciences	11 (5.1%)	185	Q1 (5.1)	151	10	Journal of cerebral blood flow and metabolism	830	Q1 (4.9)	177

### 3.6 Authors and co-cited authors analysis

**Figure 7:**
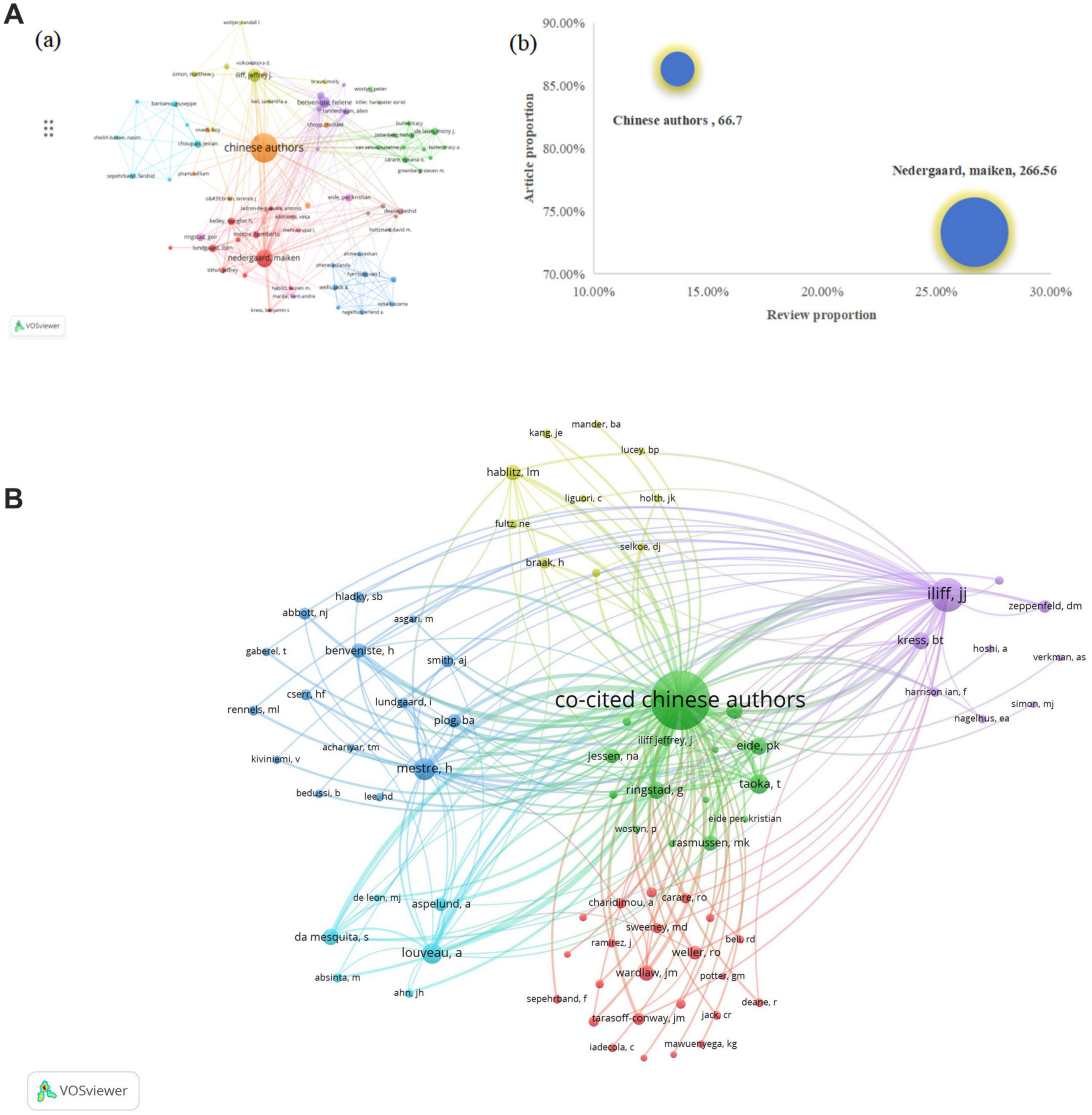
**(A) (a)** A VOSviewer-generated mapping framework depicting authors contributing to glymphatic system investigations in NDs; **(b)** Comparative analysis of publication impact between high-productivity Chinese authors and the Nedergaard M (bubble chart: X, review proportion; Y, article proportion; bubble size, citations per paper); **(B)** A VOSviewer-generated mapping framework depicting co-authors contributing to glymphatic system investigations in NDs.

Co-cited authors refer to two or more authors being simultaneously cited by others or multiple papers, establishing a co-citation relationship. In Figures 7a,B, each node represents an author or co-cited author, with node size proportional to the number of publications or citation frequency. The connecting lines between nodes indicate the degree of collaboration between authors or co-cited authors, where thicker lines denote stronger collaborative ties. Nodes sharing the same color form a cluster. Denser and more extensive connections between two clusters signify closer collaboration. As shown in Figures 7a,B, authors and co-cited authors are distributed across several distinct clusters, with those within the same cluster demonstrating closer relationships. Furthermore, we first grouped all Chinese authors into a single “Chinese authors group,” which includes authors from an internally well-known Chinese research group. Utilizing VOSviewer, we incorporated 153 authors with more than three published articles into the authors’ network. Analysis of the top 20 most influential authors worldwide revealed that the Chinese authors (351 articles, 61.69%, 23,411 citations) ranked first in terms of publication count, citation frequency, and total link strength. However, deeper examination of their academic output structure shows a predominant focus on original articles (86.32%), with review articles constituting only 13.68%. This output pattern contrasts sharply with their impact metrics: despite commanding a substantial lead in total output, their citations per paper stand at just 66.70. In comparison, Denmark’s Nedergaard M., while producing a smaller total output (45 articles), exhibits exceptional academic influence. With 11,995 cumulative citations and a total link strength of 5,111, their citations per paper reach 266.56, which is four times that of the Chinese authors. Notably, U. S. scholar Rashid Deane leads globally with 920.17 citations per paper, while the Nedergaard M.’s impact remains remarkable. Further analysis reveals the Nedergaard M. maintains a more balanced publication portfolio, with review articles accounting for 26.67% of their output—nearly double the Chinese authors’ proportion (Table 4). These differences are visually captured in the bubble chart (Figures 7A,b): the Chinese authors cluster in the “high-original/low-impact” quadrant (coordinates: X = 13.68%, Y = 86.32%), while the Nedergaard M. occupies the “balanced/high-impact” region (coordinates: X = 26.67%, Y = 73.33%). The bubble area (representing citations per paper) shows a four-fold difference between teams. Table 4 multidimensional comparative analysis quantifies these findings: the Chinese authors exemplify a “quantity-driven/original-focused” model, whereas the Nedergaard M. embodies a “quality-first/balanced-development” approach. To further analyze the situation of co-cited authors, we also combined all cited Chinese authors as “Co-cited Chinese authors.” A total of 79 authors who had been cited more than 50 times were selected to construct the co-citation network, from which the top 20 most frequently co-cited authors were identified for cluster analysis. The results indicated that all of these authors had been cited more than 150 citations. In Asia, the “Co-cited Chinese authors” had the highest total citations (3,271 citations) and the highest total link strength (112,789 TLS). Notably, Iliff, J. J. from the United States was the most frequently co-cited author (1,108 citations) with the highest total link strength (33,911 TLS), highlighting his significant academic impact and collaborative ties in the study of the glymphatic system in NDs (Table 5).

**Table 4:** Top 20 authors and collaborative citations on glymphatic system in NDs.

Continents	Author	Country	Counts	Citations	Citations per paper	Original article	Review article	Total link strength
Asia (390,68.54%)	Chinese authors	China	351 (61.69%)	23,411	66.70	303 (86.32%)	48 (13.68%)	26,309
	Taoka, Toshiaki	Japan	12 (2.11%)	1,006	83.83	7 (58.33%)	5 (41.67%)	1,149
	Lee, Hedok	South Korea	9 (1.58%)	1,602	178.00	6 (66.67%)	3 (33.33%)	906
	Naganawa, Shinji	Japan	9 (1.58%)	838	93.11	4 (44.44%)	5 (55.55%)	891
	Choupan, Jeiran	Iran	9 (1.58%)	78	8.67	8 (88.89%)	1 (11.11%)	372
North America (98,17.22%)	Iliff, Jeffrey J.	United States	25 (4.39%)	7,234	289.36	19 (76.00%)	6 (24.00%)	3,173
	Benveniste, Helene	United States	18 (3.16%)	5,854	325.22	13 (72.22%)	5 (27.78%)	2,325
	De Leon, Mony J.	United States	8 (1.41%)	93	11.63	6 (75.00%)	2 (25.00%)	614
	Kelley, Douglas H.	United States	8 (1.41%)	291	36.38	6 (75.00%)	2 (25.00%)	490
	Mestre, Humberto	United States	8 (1.41%)	2,647	330.88	4 (50.00%)	4 (50.00%)	1,390
	Tannenbaum, Allen	United States	7 (1.23%)	780	111.43	3 (42.86%)	4 (57.14%)	618
	Chopp, Michael	United States	6 (1.05%)	36	6.00	4 (66.67%)	2 (33.33%)	163
	Claassen, Daniel o.	United States	6 (1.05%)	147	24.50	6 (100.00%)	0 (0.00%)	244
	Deane, Rashid	United States	6 (1.05%)	5,521	920.17	6 (100.00%)	0 (0.00%)	2025
	Donahue, Manus J.	United States	6 (1.05%)	147	24.50	6 (100.00%)	0 (0.00%)	244
Europe (81,14.24%)	Nedergaard, Maiken	Denmark	45 (7.91%)	11,995	266.56	33 (73.33%)	12 (26.67%)	5,111
	Eide, per Kristian	Norway	10 (1.76%)	673	67.30	8 (80.00%)	2 (20.00%)	570
	Ringstad, Geir	Norway	10 (1.76%)	665	66.50	7 (70.00%)	3 (30.00%)	687
	Lundgaard, Iben	Denmark	9 (1.58%)	1867	207.44	5 (55.56%)	4 (44.44%)	910
	Wostyn, Peter	Belgium	7 (1.23%)	0	0.00	4 (57.14%)	3 (42.86%)	143

**Table 5:** Top 20 co-cited authors and collaborative citations on glymphatic system in NDs.

Continents	Co-cited author	country	Citations	Total link strength (TLS)
Asia (3,784,42.53%)	Co-cited Chinese authors	China	3,271 (36.77%)	112,789
	Taoka, T.	Japan	353 (3.97%)	12,545
	Naganawa, S.	Japan	160 (1.80%)	7,426
North America (2,663,29.93%)	Iliff, J. J.	United States	1,108 (12.45%)	33,911
	Louveau, A.	United States	392 (4.41%)	14,021
	Kress, B. T.	United States	276 (3.10%)	9,602
	Da mesquita, S.	United States	259 (2.91%)	9,506
	Hablitz, L. M.	United States	233 (2.62%)	8,831
	Benveniste, H.	United States	204 (2.29%)	7,948
	Plog, B. A.	United States	191 (2.15%)	7,621
Europe (2,450,27.54%)	Mestre, H.	Norway	475 (5.34%)	18,183
	Ringstad, G.	Norway	288 (3.24%)	11,636
	Eide, P. K.	Norway	277 (3.11%)	12,562
	Nedergaard, M.	Denmark	244 (2.74%)	7,982
	Rasmussen, M. K.	Denmark	223 (2.51%)	7,395
	Wardlaw, J. M.	United Kingdom	219 (2.46%)	7,116
	Jessen, N. A.	Norway	212 (2.38%)	6,509
	Weller, R. O.	United Kingdom	186 (2.09%)	6,680
	Aspelund, A.	Norway	174 (1.96%)	6,616
	Zeppenfeld, D. M.	Germany	152 (1.71%)	5,020

### 3.7 Co-occurrence of keywords analysis

**Figure 8:**
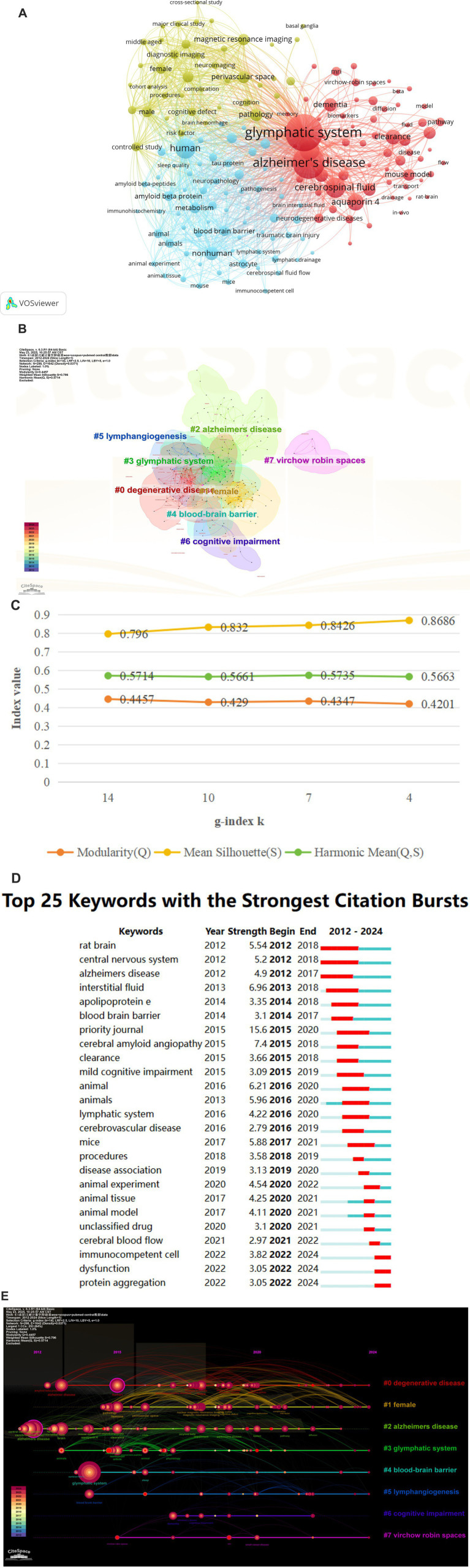
**(A)** The Co-occurrence analysis of all key words by VOSviewer (a keyword with ≥15 occurences, 170 keywords in total); **(B)** The cluster analysis of the keywords using CiteSpace; **(C)** Impact of varying g-index k on clustering quality indicators Q and S; **(D)** Top 25 keywords with the strongest ciatation bursts by CiteSpace; **(E)** CiteSpace visualization map of timeline viewer related to glymphatic system in NDs.

Examining lexical co-occurrence, thematic clustering, and temporal evolution serves as a methodological approach to identify trending themes with rapidly evolving prominence. Visualization of these lexical patterns enables researchers to elucidate core priorities and intellectual trends within the discipline, supporting holistic comprehension of its evolutionary pathways. From a corpus of 865 publications, 5,346 distinct keywords were systematically identified through VOSviewer’s analytical framework. Since some words, such as “Alzheimer’s disease” and “Alzheime’s disease,” expressed the same meaning, these synonyms were combined in the analysis. The top 20 most frequent keywords are displayed in Table 6. The keyword analysis identified “Glymphatic system” (695 occurrences) and “Alzheimer’s disease” (472 occurrences) as the highest-frequency terms, with subsequent prominence observed for “Human” (*n* = 217) “Cerebrospinal-fluid” (*n* = 215) and “brain” (*n* = 213). These lexical clusters delineate primary research frontiers in glymphatic pathway investigations related to NDs. Lexical network mapping via VOSviewer (Figure 8A) employed two normalization criteria: frequency (≥15 occurrences) and semantic merging of equivalent terms, ultimately yielding a refined lexicon of 170 terms. Clustering of the network map based on keyword co-occurrence analysis was performed to reflect the basic knowledge structure of related research areas. CiteSpace software was used to cluster the keywords in the literature. This study utilized CiteSpace software and the log-likelihood ratio (LLR) method to cluster keywords from the literature, selecting g-index k = 14 (the maximum value) to ensure high confidence and reduce noise. Under this strict threshold, 8 clusters were generated, while core themes remained clear (Figure 8B). The clustering modularity (Q) value was 0.4457, and the mean silhouette (S) was 0.796, indicating significant clustering structure (Q > 0.3) and efficient, credible clustering (S > 0.7). To assess robustness, we examined changes in Q, S, and Harmonic Mean (Q, S) across different k values (Figure 8C). As the k value decreased from 14 to 4, the number of clusters reduced, yet changes in Q and S stayed below 10%. Harmonic Mean (Q, S) remained stable, suggesting the overall clustering quality was consistent across various k values. Although the Harmonic Mean (Q, S) was below 0.6, the slight variations in Q and S demonstrate the robustness of the clustering results. The eight cluster labels are as follows: neurodegenerative diseases (#0), female (#1), Alzheimer’s disease (#2), brain lymphatic system (#3), blood–brain barrier (#4), lymphangiogenesis (#5), cognitive impairment (#6), and Virchow-Robin spaces (#7), detailed in Table 7. The stability of core clustering themes further supports the robustness of the clustering results. When the frequency of a particular keyword increases sharply within a certain period, it is referred to as a burst keyword. This phenomenon provides important insights for identifying emerging research topics and hotspots in a given field (Hou et al., 2023). Figure 8D displayed the top 25 keywords with the strongest citation bursts. The keyword with the highest citation burst was priority journal (15.6) from 2015 to 2020, followed by cerebral amyloid angiopathy (7.4) and interstitial fluid (6.96). The keywords with the longest duration of the strongest citation bursts were rat brain and central nervous system from 2012 to 2018. A timeline viewer for these keywords was constructed using CiteSpace. The timeline visualization categorizes keywords through temporal stratification, enabling chronological tracking of topic-specific developments across distinct research phases. Figure 8E illustrates the intellectual priorities and developmental pathways of glymphatic system studies in NDs.

**Table 6:** The top 20 keywords related to glymphatic system in NDs.

Rank	Keywords	Occurrences	Rank	Keywords	Occurrences
1	Glymphatic system	695	11	Pathology	100
2	Alzheimer’s disease	472	12	Interstitial fluid	100
3	Human	217	13	Impairment	91
4	Cerebrospinal-fluid	215	14	Mouse model	87
5	Brain	213	15	Magnetic resonance imaging	83
6	Aquaporin 4	161	16	Amyloid-beta	82
7	Clearance	128	17	Male	80
8	Nonhuman	114	18	Nuclear magnetic resonance imaging	80
9	Sleep	110	19	Metabolism	78
10	Dementia	107	20	Neurodegeneration	71

**Table 7:** Cluster analysis results of glymphatic system research in NDs.

ClusterID	Silhouette	mean (Year)	Label (LSI)	Label (LLR)	Label (MI)
0	0.618	2019	Glymphatic system; central nervous system disease; mood disorder; perivascular space; transcription factor | s disease; endothelial cells; substantia nigra; gut microbiota; reactive oxygen speciess	Degenerative disease (16.66, 1.0E-4); female (13.78, 0.001); microglia (13.77, 0.001); male (13.08, 0.001); stem cell niche (11.02, 0.001)	Bacterial infection (0.94); 3 d cultures (0.94); c
1	0.83	2020	Magnetic resonance; systolic blood; apparent diffusion; diastolic blood; community dwelling | glymphatic system; magnetic resonance imaging; perivascular spaces; robin spaces; paravascular spaces	Female (37.71, 1.0E-4); male (32.65, 1.0E-4); aged (29.73, 1.0E-4); middle aged (27.66, 1.0E-4); longitudinal study (17.78, 1.0E-4)	18f-florzolotau (1.01); clearance syste
2	0.874	2015	Glymphatic system; perivascular spaces; choroid plexus; dural lymphatics; glymphatic pathways | alzheimers disease; 4d flow mri; mean blood flow; pulsatility index; brain tumorigenesis	Alzheimers disease (70.79, 1.0E-4); interstitial fluid (27.52, 1.0E-4); clearance (25.86, 1.0E-4); aqp4 (23.42, 1.0E-4); mouse model (23.19, 1.0E-4)	Autoimmunity (1.17); chronic pain (1.17); coffee p
3	0.843	2017	Cerebrospinal fluid; glymphatic pathway; perivascular space; animal models; glucocorticoid receptor | amyloid beta; blood brain; nuclear magnetic resonance; endothelial nitric oxide; beta adrenergic receptor	Alzheimers disease (12.17, 0.001); glymphatic system (10.5, 0.005); animal model (8.92, 0.005); mouse (8.92, 0.005); mice (8.92, 0.005)	Cell transport (0.7); cerebellum (0.7); chemistry
4	0.828	2016	Glymphatic system; cerebrospinal fluid; waste clearance; transgenic mouse; sleep disorders | alzheimers disease; blood–brain barrier; reactive astrocytes; hyaluronic acid; plga nanoparticles	Glymphatic system (25.93, 1.0E-4); blood–brain barrier (18.92, 1.0E-4); sleep (15.71, 1.0E-4); sleep deprivation (15.53, 1.0E-4); glymphatic (11.8, 0.001)	cerebrovascular activity (0.98); brain ventricles
5	0.781	2020	Glymphatic system; perivascular spaces; paravascular spaces; magnetic resonance imaging; brain cysts | cerebrospinal fluid; blood brain; chronic kidney; renal replacement; nervous system	Alzheimers disease (12.71, 0.001); lymphangiogenesis (9.37, 0.005); glioma (9.37, 0.005); subarachnoid hemorrhage (9.37, 0.005); lymphatic drainage (8.46, 0.005)	Neuroinvasive pathways (0.52); hydrocephalus (0.52)
6	0.957	2019	Glymphatic system; parkinsons disease; lewy bodies dementia; multisystem atrophy; circadian blood pressure patterns | s disease; sleep disorders; tau proteins; melatonin levels; cholinesterase inhibitors	cognitive impairment (23.16, 1.0E-4); diagnosis (11.92, 0.001); alzheimer disease (11.66, 0.001); alzheimer’s disease (11.2, 0.001); dementia (8.69, 0.005)	cadherins (0.24); transgenic mice (0.24); neurosci
7	0.983	2019	Glymphatic system; cerebral small vessel disease; enlarged perivascular spaces; impaired cognition; capillary rarefaction | perivascular space; multiple sclerosis; csf shunting; csf dynamic disturbances; glymphatic circulation	Virchow robin spaces (22.41, 1.0E-4); small vessel disease (15.83, 1.0E-4); blood brain barrier (bbb) (14.93, 0.001); perivascular space (pvs) (14.93, 0.001); cerebral small vessel disease (10.27, 0.005)	Spectral analysis (0.1); Glucocerebrosidase (0.1)

### 3.8 Tripartite network analysis of author-keyword-journal interconnections

**Figure 9:**
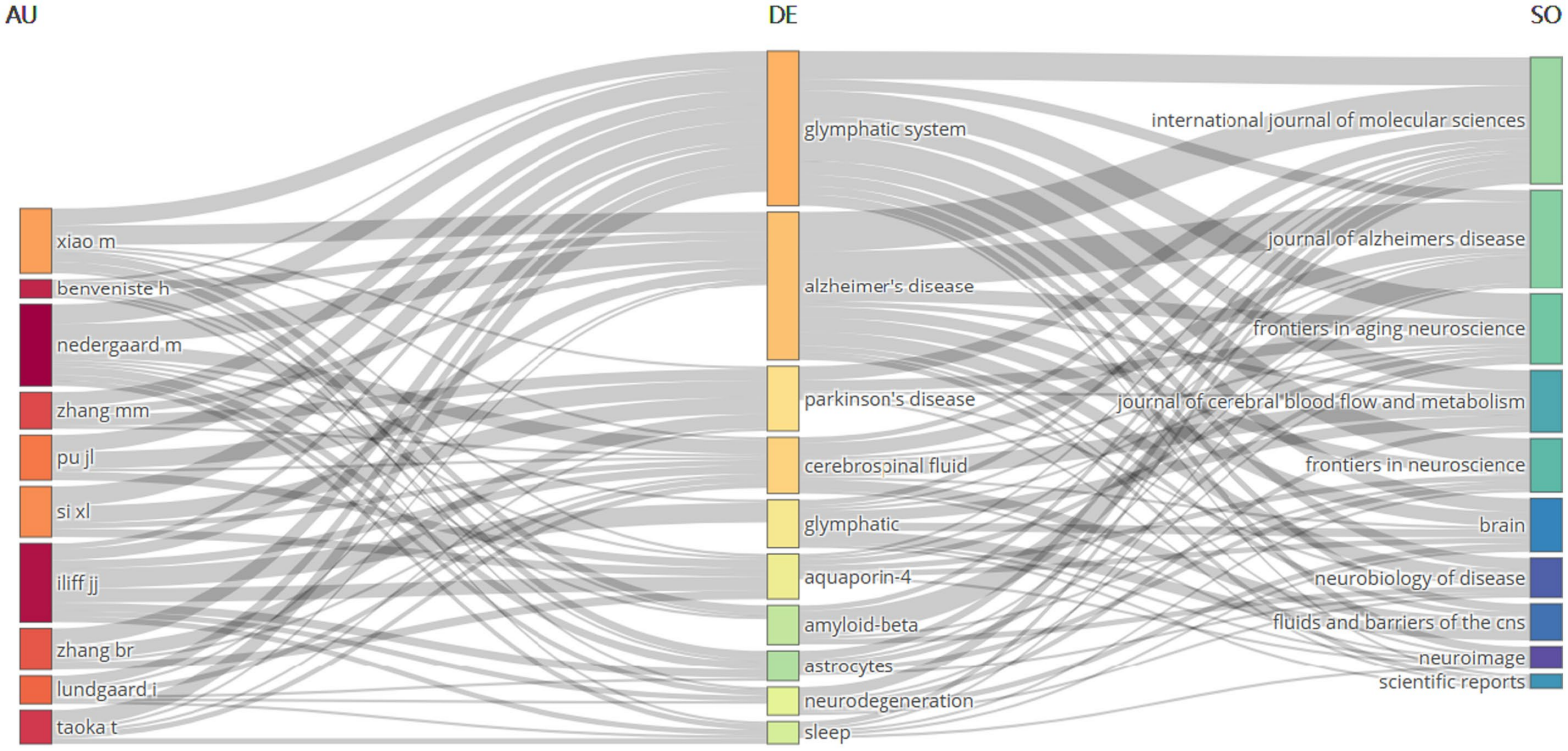
Tripartite network visualization of author-keyword-journal relationships.

The tripartite network visualization methodology facilitates systematic integration and examination of multidimensional relationships between bibliometric entities through the construction of interconnected node-edge mappings. Applying this three-dimensional network analysis framework to bibliometric data, we established three interconnected domains: “Author” (left panel), “keyword” (central panel), and “journal” (right panel), generating an integrated “author-keyword-journal” knowledge architecture as visualized in Figure 9. The analysis reveals that the research hotspots are primarily concentrated in two core areas: the “Glymphatic system” and “Neurodegenerative diseases” (with a particular focus on “Alzheimer’s disease” and “Parkinson’s disease”). This highlights the significance and intrinsic connection between these two research directions. Additionally, high-frequency keywords also involve cutting-edge topics such as “cerebrospinal fluid,” “aquaporin-4,” “*β*-amyloid,” and “sleep.” In the co-occurrence network analysis of authors and keywords, the research of Professors Nedergaard M. and Xiao M. is significantly associated with both the “glymphatic system” and “Alzheimer’s disease.” In contrast, Professor Iliff J. J. has focused his research on the relationship between “Aquaporin-4” and the Glymphatic system. The journal-keyword analysis indicates that studies on the “glymphatic system” are mainly published in three authoritative journals: *International Journal of Molecular Sciences, Journal of Cerebral Blood Flow and Metabolism, and Frontiers in Aging Neuroscience*. Research on “Alzheimer’s disease” exhibits a bimodal distribution in *International Journal of Molecular Sciences* and *Journal of Alzheimer’s Disease.* Studies related to “cerebrospinal fluid” are predominantly featured in *Journal of Cerebral Blood Flow and Metabolism*. Through this network topology analysis, not only have the influential core scholars in the field and their research directions been clearly identified, but also the important status of *International Journal of Molecular Sciences*, *Journal of Alzheimer’s Disease*, and *Frontiers in Aging Neuroscience* as key academic dissemination platforms driving the development of this discipline has been established.

### 3.9 Co-cited references and reference bursts analysis

**Figure 10:**
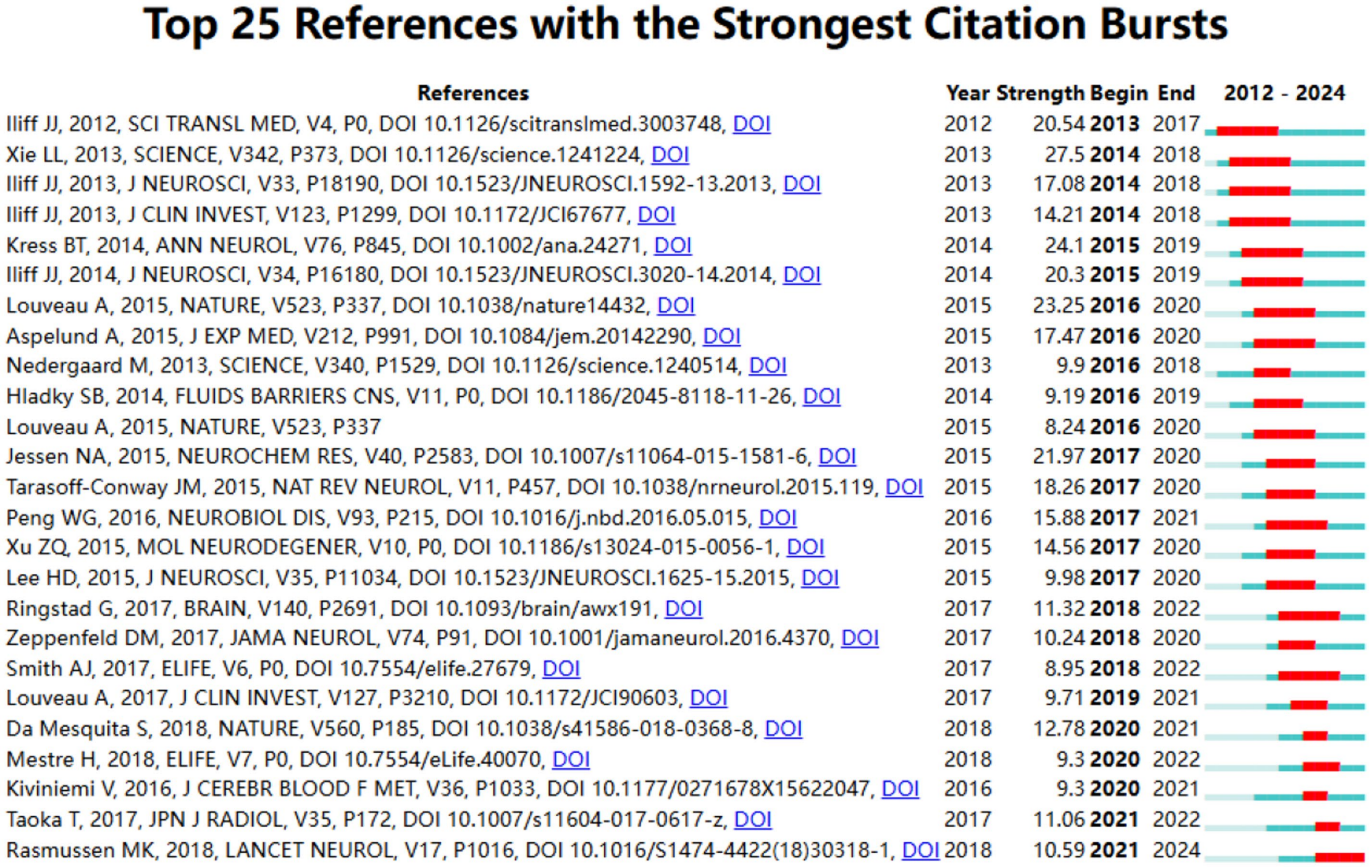
Citations burst dynamics: top 25 references analysis.

CiteSpace was utilized to identify the top ten most frequently cited references related to the glymphatic system in NDs (Table 8). Each of these ten references has received 65 or more citations. Notably, the article ranked first is “Glymphatic failure as a final common pathway to dementia” by Nedergaard and Goldman (2020) cited 106 times; the second is “Impaired glymphatic function and clearance of tau in an Alzheimer’s disease model” by Harrison et al. (2020) cited 95 times; and the third is “The glymphatic pathway in neurological disorders” by Rasmussen MK et al., cited 93 times. From the citation counts of the top three articles, it is evident that the pathways and functions of the glymphatic system play a crucial role in the onset and progression of NDs. The analysis of citation bursts among references is a powerful tool for pinpointing documents that have garnered a sudden increase in citations over a particular timeframe. This not only highlights research hotspots but also delineates prevailing trends. It serves as a crucial guide for uncovering nascent themes and key research concentrations within a given domain. In this study, a total of 25 references with the most pronounced citation bursts were detected, as depicted in Figure 10. The top three most cited references are titled “Sleep Drives Metabolite Clearance from the Adult Brain” (Burst Strength: 27.5; published in 2013), “Impairment of paravascular clearance pathways in the Aging Brain” (Burst Strength: 24.1; published in 2014), and “Structural and functional features of central nervous system lymphatic vessels” (Burst Strength: 23.25; published in 2015). Figure 10 displays that there are 12 articles with a citation period of more than 4 years, indicating that these articles have garnered more sustained attention. One article is currently still in the period of citation burst, which can reflect, to some extent, the developmental direction of glymphatic system research in NDs at present and in the future.

**Table 8:** The top 10 co-cited references related to glymphatic system in NDs.

Rank	Year	Author	Title	Journal	Citation
1	2020	Nedergaard M.	Glymphatic failure as a final common pathway to dementia	Science	106
2	2020	Harrison Ian F.	Impaired glymphatic function and clearance of tau in an Alzheimer’s disease model	Brain	95
3	2018	Rasmussen M. K.	The glymphatic pathway in neurological disorders	Lancet Neurol	93
4	2018	Mestre H.	Aquaporin-4-dependent glymphatic solute transport in the rodent brain	Elife	87
5	2019	Zou W. Y.	Blocking meningeal lymphatic drainage aggravates Parkinson’s disease-like pathology in mice overexpressing mutated α-synuclein	Transl Neurodegener	76
6	2018	Da Mesquita S.	Functional aspects of meningeal lymphatics in ageing and Alzheimer’s disease	Nature	75
7	2020	Hablitz L. M.	Circadian control of brain glymphatic and lymphatic fluid flow	Nat commun	71
8	2017	Zeppenfeld D. M.	Association of Perivascular Localization of Aquaporin-4 With Cognition and Alzheimer Disease in Aging Brains	JAMA Neurol	67
9	2018	Mestre H.	Flow of cerebrospinal fluid is driven by arterial pulsations and is reduced in hypertension	Nat commun	65
10	2019	Hablitz L. M.	Increased glymphatic influx is correlated with high EEG delta power and low heart rate in mice under anesthesia	Sci Adv	65

## 4 Discussion

### 4.1 Global research trends

This study employs scientometric visualization tools to map temporal dynamics and intellectual focal points in glymphatic system research associated with NDs across the 2012–2024 period. Our findings reveal that during the initial period from 2012 to 2016, the publication volume remained relatively limited, yet this stage established essential groundwork for deciphering the glymphatic system’s role in NDs. The early developmental phase spanning 2017 to 2019 saw gradual growth in scholarly output, while the rapid expansion phase from 2020 to 2023 experienced a substantial rise in literature, signaling heightened academic interest. By 2024, annual publications surged to 233, representing a 1.47-fold increase compared to 2023, a trajectory suggesting sustained growth and cementing the glymphatic system as a pivotal focus for future NDs investigations. In terms of publication type structure, original articles dominate among the high-quality literature collected (accounting for approximately 61.39%), while review articles account for 38.61%. This structural feature indicates that the field is primarily driven by empirical and mechanistic investigations. However, as research continues to deepen, the number of review articles has gradually increased, providing a theoretical foundation and directional guidance for knowledge integration, frontier advancement, and paradigm shifts within the field. Overall, research on the glymphatic system and NDs has evolved from the initial stage of theoretical emergence and basic exploration to a period characterized by mechanistic refinement and comprehensive synthesis. In the future, with the continued accumulation of original research achievements and the ongoing emergence of high-impact review articles, this field is expected to accelerate toward a new stage marked by interdisciplinary integration, translational application, and theoretical leadership.

Research priorities center on foundational mechanisms, clinical translation, and imaging evaluation, with neuroscience accounting for the largest thematic share. Temporally, publication trends exhibit both chronological evolution and geographic-institutional stratification. From a temporal perspective, the publication trends not only reflect chronological evolution but also reveal distinct patterns of geographic and institutional stratification. Globally, research output is predominantly concentrated in four major regions: Asia, Europe, North America, and Australia. Among these, China and the United States lead in both publication volume and citation impact, followed by Italy and Japan. In terms of institutional contributions, Chinese universities account for the highest overall output (109 articles, 30.70%), with institutions from the United States and Denmark also ranking among the top performers. Notably, the University of Rochester in the United States stands out globally for both the number of publications and citation influence, further consolidating the country’s leading position in this field. Leading universities from Denmark and Japan have also demonstrated exceptional academic performance. Chinese universities maintain close collaborations with research institutions in other countries, underscoring China’s rapid progress and growing influence in the international academic community. However, some institutions, such as Massachusetts General Hospital and Eindhoven University of Technology in the Netherlands, have not yet established close cooperative relationships with core research institutions in China, the United States, and other major countries. Therefore, strengthening academic exchange and international collaboration is essential for advancing research on the glymphatic system and NDs worldwide. Table 3 presents the top 10 core journals related to glymphatic system and NDs research. Most of these journals are classified as JCR Q1, highlighting the strong academic impact and global standing of research in this area. Similarly, the majority of the top 10 most-cited journals also belong to JCR Q1, including well-regarded journals such as *Brain*, *Journal of Cerebral Blood Flow and Metabolism*, and *Neurobiology of Disease*. This not only demonstrates the high recognition that glymphatic system research has earned from the academic community, but also further confirms the significance of these journals within the field. Many high-quality, high-impact journals have shown a strong interest in this area, providing strong support for the dissemination and development of related research. Overall, our research findings will help researchers quickly and accurately identify suitable journals to obtain the latest advancements or publish articles in the field of the glymphatic system and NDs. Combined with the summary of the glymphatic system and NDs research field in Figure 3 and the dual map overlay of journals in Figure 6C, it is evident that the system is currently shifting towards clinical translation and updates in imaging techniques. In addition, a systematic analysis of core authors and their co-citation networks in the global research field of the glymphatic system and NDs reveals that Chinese authors rank first worldwide in both the number of publications and total citations. However, their academic output is dominated by original articles, with a relatively low proportion of review papers, and the citation per paper is significantly lower than that of top teams from developed countries such as Denmark. For instance, although the total number of publications from the Nedergaard M. is less than that of the group of Chinese authors, their output features a more balanced structure and a higher proportion of review papers, resulting in an impressive average citation count of 266.56 per paper, which highlights a “quality-first and balanced development” research strategy. Further analysis of the collaboration network shows that, although Chinese authors demonstrate strong cohesion regionally, in-depth collaboration with leading international teams—particularly those represented by Iliff from the United States—remains insufficient. Therefore, it is recommended that Chinese authors, while continuing to strengthen their capacity for original research, further optimize their academic output structure, enhance the innovativeness and international impact of their research, and actively pursue high-level international collaborations. Such efforts will facilitate a shift from “quantity-driven” to “quality-led” development in the field. This strategic adjustment is of great significance for promoting theoretical innovation and achieving major breakthroughs in global research on the glymphatic system in NDs. Nevertheless, continuously improving the scientific rigor of research methods and deepening theoretical insight remain key to future academic advancement. At the same time, establishing cross-institutional collaborative networks and fostering synergistic innovation will be important strategies for driving paradigm shifts and realizing significant research progress.

### 4.2 Knowledge base

Co-citation analysis constitutes a rigorous methodological framework for quantifying conceptual linkages between scholarly publications. Frequent citation patterns typically denote seminal conceptual frameworks within a research domain, while primary investigations serve as critical vectors for disciplinary innovation. Our scientometric evaluation of 865 peer-reviewed studies addressing glymphatic-neurodegenerative interactions employed citation frequency thresholds to identify ten paradigm-defining works. The review “Glymphatic failure as a final common pathway to dementia” by Nedergaard and Goldman (2020) published in *Science* in 2020 (cited 106 times), is the most frequently cited paper in this field. The authors propose that dysfunction of the glymphatic system may be a common pathological mechanism underlying various dementias, including Alzheimer’s disease and vascular dementia. The article summarizes both basic and clinical research, noting that with age, the glymphatic system’s ability to clear brain metabolic waste declines, leading to the accumulation of harmful proteins such as Aβ and tau, which results in neuronal damage and cognitive decline. Sleep disturbances, cardiovascular risks like hypertension, and trauma can accelerate this process. Early improvement of glymphatic function is considered important for dementia prevention. The second highly cited article is “Impaired glymphatic function and clearance of tau in an Alzheimer’s disease model” by Harrison et al. (2020) published in *Brain* in 2020. This study, using a P301S mutant tau mouse model, found that the glymphatic system’s clearance of tau was reduced by 40% compared to normal mice, closely associated with the loss of astrocytic AQP4 polarity. This leads to abnormal tau accumulation and neuronal loss, directly confirming the key link between the glymphatic system and tau pathology. The third article, published by Rasmussen et al. (2018) in *The Lancet Neurology* in 2018, systematically reviews the role of the glymphatic system in Alzheimer’s disease, traumatic brain injury (TBI), stroke, and migraine. Glymphatic dysfunction is a common feature in these disorders; for example, reduced clearance in traumatic brain injury (TBI) leads to brain edema and accumulation of inflammatory products, while stagnant glymphatic flow during stroke worsens neuronal damage, highlighting its broad pathological significance. The fourth article, “Aquaporin-4-dependent glymphatic solute transport in the rodent brain” by Mestre et al. (2018), compared AQP4 knockout mice with wild-type mice and showed that AQP4 deficiency significantly reduces glymphatic flow speed and clearance efficiency, and increases Aβ deposition, underscoring the central role of AQP4 in glymphatic transport. The fifth article by Zou et al. (2019) published in *Translational Neurodegeneration* in 2019, showed that blocking meningeal lymphatic drainage in a Parkinson’s disease mouse model led to increased aggregation of *α*-synuclein, greater loss of dopaminergic neurons in the substantia nigra, and worsened motor deficits, along with increased infiltration of peripheral immune cells into the brain. This suggests that the meningeal lymphatic system is involved in both the clearance of harmful proteins and the regulation of inflammation, playing an important role in Parkinson’s disease progression. The sixth article by Da Mesquita et al. (2018), published in *Nature* in 2018, found that aging and AD model mice exhibit degeneration and dysfunction of meningeal lymphatic vessels and reduced Aβ clearance in the brain. Promoting lymphangiogenesis improved these indicators, indicating that degeneration of meningeal lymphatics is a key factor in AD-related protein accumulation. The seventh article by Hablitz et al. (2020) published in *Nature Communications* in 2020, was the first to show that glymphatic and meningeal lymphatic flow rates in mice have a clear day-night rhythm, being higher at night than during the day. Disruption of circadian rhythms lowers Aβ clearance, suggesting that sleep and circadian rhythms are important for glymphatic function and brain health. The eighth article by Zeppenfeld et al. (2017) published in *JAMA Neurology* in 2017, analyzed postmortem brain tissue and found the loss of perivascular AQP4 polarity was much greater in AD patients than in controls and was associated with the Apolipoprotein E epsilon 4 allele (APOE ε4) genotype, indicating that AQP4 localization is closely related to glymphatic function and cognitive impairment in AD. The ninth article by Mestre et al. (2018) published in *Nature Communications* in 2018, showed that hypertension reduces arterial pulsatility and cerebrospinal fluid flow, decreases Aβ clearance, and impairs spatial memory, while antihypertensive treatment can partially reverse these effects. This highlights the importance of blood pressure control in preventing cognitive impairment. The tenth article by Hablitz et al. (2019) published in *Science Advances* in 2019, demonstrated that under anesthesia, increased electroencephalogram delta waves and low heart rate enhance glymphatic flow in mice, which is associated with expansion of the interstitial space. This state mimics glymphatic activation during deep sleep and provides a theoretical basis for non-invasively modulating glymphatic function.

Overall, the ten most highly co-cited references based on annual average citations have not only laid a solid theoretical foundation for understanding the mechanistic link between NDs and the glymphatic system, but have also accelerated the translation of basic research into clinical applications. Published in JCR Q1 journals within the field, these high-impact articles—including comprehensive systematic reviews and pioneering empirical studies—primarily focus on the fundamental mechanisms of the glymphatic system in NDs, key discoveries using animal models, and early clinical evidence. Collectively, these works have played a leading role in elucidating the relationship between NDs and the glymphatic system.

### 4.3 Research hot spots and frontiers

Keyword analysis as an important tool for evaluating research hotspots and the evolution of scientific disciplines has revealed the current cutting-edge topics in the field of NDs for this study. A systematic review of high-frequency keywords such as “glymphatic system” “Alzheimer’s disease” “cerebrospinal fluid” “Aquaporin 4” “clearance” “sleep” and “pathology” demonstrates that current academic attention is primarily focused on the role of the glymphatic system in regulating cerebrospinal fluid dynamics metabolic waste clearance AQP4-related mechanisms and regulatory factors such as sleep—especially with regard to the specific mechanisms in major diseases like AD. Further keyword clustering analysis (Figure 8B; Table 7) more intuitively delineates the research directions and main connotations in this field. Specifically the eight clusters cover NDs as a whole (Cluster 0) sex-related factors (Cluster 1) Alzheimer’s disease (Cluster 2) glymphatic system mechanisms (Cluster 3) blood–brain barrier (Cluster 4) lymphangiogenesis (Cluster 5) cognitive impairment (Cluster 6) and specific cerebral structures such as Virchow-Robin spaces (Cluster 7). These clusters encompass research subjects regulatory factors mechanisms of action and also include specific anatomical structures and physiological pathways. Based on the above analysis future research should further focus on the following specific directions:

#### 4.3.1 Mechanistic insights into the glymphatic system’s role in NDs pathology

The glymphatic system is now recognized as the primary pathway for clearing metabolic waste and neurotoxic proteins—such as Aβ and tau—from the brain, and significant clinical and preclinical evidence has linked its dysfunction to the onset and progression of NDs, including AD and PD (Rehman et al., 2024; Beschorner and Nedergaard, 2024). Advances such as functional MRI have enabled noninvasive assessment of glymphatic activity, revealing that both cerebrospinal fluid clearance and glymphatic flow decline with age, which correlates with increased brain aging and vulnerability to disease (Malis et al., 2024; Naganawa et al., 2024). Nonetheless, critical questions remain—particularly regarding the molecular underpinnings, dynamic regulation, and cooperative interactions between the glymphatic system, meningeal lymphatics, and other clearance pathways. Some researchers also emphasize anatomical and functional heterogeneity between human and animal models, which further complicates mechanistic conclusions (Jessen et al., 2015; Mestre et al., 2020). Extensive animal and translational studies demonstrate that impaired glymphatic function not only blocks the efflux of Aβ, tau, and other toxic proteins—leading to their aberrant accumulation—but also exacerbates neuroinflammation and neuronal loss, thus advancing cognitive decline and acting as a final common pathway for diseases such as AD and PD (Boland et al., 2018; Rasmussen et al., 2018; Nedergaard and Goldman, 2020; Ding et al., 2021; Ishida et al., 2022). Importantly, modifiable factors like exercise, especially high-intensity interval training, have been shown to enhance astrocytic AQP4 polarization and boost glymphatic clearance, whereas sleep disturbances substantially impair glymphatic activity and independently increase neurodegenerative risk (Feng et al., 2023; Gottesman et al., 2024). Despite growing recognition of the glymphatic system’s crucial involvement in the etiology and progression of NDs, key obstacles remain, including suboptimal imaging sensitivity, standardization issues, discrepancies between animal and human studies, limited mechanistic depth, and the uncertain clinical efficacy of targeted interventions. Moving forward, the field stands to benefit from large-scale human studies, innovative dynamic molecular imaging, and multicenter interventional trials to further advance foundational understanding and enable effective clinical translation.

#### 4.3.2 The core role of AQP4 and its polarization in cerebrospinal fluid flow and clearance function

AQP4 is highly polarized and distributed at the perivascular end-feet of astrocytes, serving as the molecular basis for the exchange between cerebrospinal fluid (CSF) and interstitial fluid (ISF), and for the efficient transport of waste via the glymphatic system (Iliff et al., 2012). A substantial body of research has demonstrated that the polarized state of AQP4 determines the efficiency with which CSF can drive the clearance of macromolecules such as Aβ, tau, and *α*-synuclein (Mestre et al., 2018; Ishida et al., 2022; Gomolka et al., 2023; Zhang et al., 2023). Genetic models have shown that both AQP4 knockout and loss of its polarization (e.g., Snta1 mutation) lead to impaired CSF-ISF exchange and waste accumulation, ultimately resulting in Aβ plaque formation, tau deposition, and exacerbation of cognitive dysfunction (Zeppenfeld et al., 2017; Simon et al., 2022; Gomolka et al., 2023). In human aging and in AD patients, loss of AQP4 polarization is highly correlated with cognitive decline, highlighting this molecular marker as a novel target for diagnosis and intervention (Zeppenfeld et al., 2017). In models of neurological diseases such as PD, impaired AQP4 repolarization further aggravates α-synuclein aggregation and inflammatory responses, suggesting its broad neuroprotective significance (Zhang et al., 2023; Si et al., 2024). The polarization of AQP4 is regulated by multiple factors, including protein complexes and matrix metalloproteinase Matrix metalloproteinase-9; targeted modulation of these pathways has been shown to partially restore glymphatic function and reverse pathological progression (Si et al., 2024). Physiological interventions such as high-intensity training and circadian rhythm synchronization can also effectively enhance AQP4 polarization and promote brain waste clearance (Hablitz et al., 2020; Feng et al., 2023). However, it is important to note that the upstream regulatory networks of AQP4 polarization remain unclear, noninvasive and dynamic clinical monitoring tools are lacking, and interdisciplinary approaches such as AI imaging and biomarker analysis have yet to be widely applied—leaving translational pathways in need of further integration (Mestre et al., 2017; Abbott et al., 2018; Rasmussen et al., 2018; Benveniste et al., 2019). Future directions should focus on elucidating the dynamic regulatory mechanisms of AQP4 polarization, developing imaging and molecular monitoring systems, and achieving precision interventions and innovations in diagnosis and therapy targeting this new molecule.

#### 4.3.3 Dialectical impact of the interaction between multidimensional regulatory factors and the coupling mechanism of the brain waste clearance system on the progression of neurological diseases

The brain waste clearance system comprises a complex, multilayered network that includes the glymphatic system, meningeal lymphatic vessels, the blood–brain barrier, and brain microstructures, and its function is regulated by the coordinated action of multiple endogenous and exogenous factors (Yankova et al., 2021). High-quality deep sleep significantly enhances the metabolic waste clearance efficiency of the glymphatic system and CSF flow; during sleep, the interstitial space between brain cells expands substantially (by approximately 60%), facilitating faster CSF circulation and more efficient clearance of solute waste products such as Aβ (with clearance efficiency approximately 2 times higher than during wakefulness) (Xie et al., 2013). Conversely, sleep disturbances not only damage the glymphatic network but also directly undermine the blood–brain barrier, leading to inflammatory activation and abnormal protein accumulation, thus serving as risk factors for NDs (Avilez-Avilez et al., 2024; Gottesman et al., 2024; Ozdinler, 2024). Similarly, aging induces an overall decline in CSF outflow and glymphatic efficiency, accompanied by expansion of perivascular spaces, microenvironmental dysregulation, and glial activation, which further impede waste clearance and provide both anatomical and molecular bases for cognitive dysfunction (Andriuta et al., 2024; Malis et al., 2024; Xiong et al., 2024). Sex differences are also reflected in the expression of relevant genes, channel functions, and disease susceptibility, but mechanistic research at present remains insufficient. Notably, there exists a complex and dynamic coupling among clearance subsystems: disorders of meningeal lymphatics and microvascular structural damage often form a vicious cycle with waste accumulation and inflammatory activation, thereby accelerating the neurodegeneration process (Cai et al., 2024; Chen et al., 2024; Wen et al., 2024; Zedde and Pascarella, 2024). Although advanced imaging techniques can dynamically scan perivascular space changes, there is still a lack of direct evidence linking these with the microstructural–functional causal chain, which limits precise risk prediction and target identification (Sacchi et al., 2024). Most existing studies are cross-sectional or single-factor investigations, lacking systematic, multimodal, dynamic, and longitudinal multi-factorial coupling models and integrated mechanism analysis. Future research should focus on synergistic analyses combining high-resolution multimodal imaging and molecular mechanisms, aiming to achieve multivariable, network-based, and precise interventions.

#### 4.3.4 Translational applications of artificial intelligence (AI) modeling, imaging analysis, and novel neuroregulation Technologies in glymphatic system functional interventions

AI modeling and advanced multimodal imaging techniques are gradually transforming both the basic research and clinical translation landscape of the glymphatic system. AI-based machine learning can deeply explore risk prediction of core factors such as AQP4 polymorphisms (Beer et al., 2024). In addition, AI-based velocimetry algorithms enable high-precision, noninvasive quantification of intracerebral cerebrospinal fluid dynamics parameters, providing a novel technical approach for elucidating brain clearance mechanisms in high-dimensional spaces and for early evaluation and classification of brain diseases such as AD (Boster et al., 2023). The new generation of multimodal MRI (such as diffusion tensor imaging and perivascular space visualization) shows significant value in early screening and dynamic monitoring of disease progression in brain disorders such as autism, helping to identify the association between glymphatic dysfunction and the evolution of disease phenotypes (Wang et al., 2023; Soares et al., 2024). It is worth emphasizing that imaging-guided, subject-specific modeling of the glymphatic system not only provides an accurate reflection of each individual’s brain clearance function, but also reveals a close association between reduced glymphatic transport efficiency and increased amyloid deposition in the brain (Johnson et al., 2023). This quantitative model offers a scientific basis for risk identification and precise intervention, and further promotes the in-depth understanding of the mechanisms underlying neurodegenerative diseases as well as individualized management. Moreover, neuroregulation techniques and Adeno-Associated Virus Serotype 1 (AAV1)-based delivery provide feasible pathways for targeted repair of the glymphatic network and gene therapy, with promising progress observed in both rat/human models and special environments (such as space) (Wu et al., 2019; Mathiesen et al., 2023; Soares et al., 2024). However, challenges such as dynamic imaging resolution, clinical standardization, multifactorial mechanism modeling, and AI interpretability remain, leaving a gap between technological advances and precise intervention (Zhang et al., 2023; Zhu et al., 2023). In the future, it will be essential to strengthen the deep integration of multicenter, large-sample, dynamic longitudinal data with AI modeling, and to promote full-chain coordination and innovation across imaging, AI, neuroregulation, and molecular mechanism domains. Addressing challenges related to standardization and interpretability is crucial to support the precise and personalized management of glymphatic function and related NDs.

In summary, the glymphatic system and its molecular networks, especially AQP4 polarization, provide new theoretical and technological foundations for the etiology and intervention of NDs. Future research should focus on: (1) optimizing and standardizing *in vivo* imaging and molecular dynamic assessment for precise quantification of glymphatic function; (2) elucidating regulatory networks and cross-species mechanisms of key molecules such as AQP4 polarization; (3) integrating multidimensional regulatory factors and waste clearance to construct macro–micro dynamic coupling models; and (4) advancing the application of AI and imaging technologies in multi-center and diverse environments to improve early screening, targeted prevention, and personalized management of NDs. We look forward to more researchers joining the field to drive breakthroughs in brain health and disease prevention.

### 4.4 Advantages and limitations

This study offers several distinct advantages. Firstly, this study is the first to conduct a scientometric analysis specifically focusing on the relationship between the glymphatic system and NDs. It provides a foundational basis and direction for systematic research in this field. Secondly, by integrating multiple scientometric methods, this study presents a comprehensive and multi-level refined analysis of research hotspots, temporal trends, and collaborative networks. This approach not only overcomes the limitations of traditional reviews, but also uncovers new potential connections between glymphatic mechanisms and NDs. Finally, we used data from Web of Science, Scopus, and PubMed Central, which significantly broadens the scope of the literature surveyed and improves the reliability and representativeness of the results.

Nevertheless, several limitations should be noted in this study. Firstly, this study only included literature published in English during the selection process, which may have resulted in the omission of important studies published in other languages. Secondly, citation counts are influenced by the time of publication, meaning that recently published studies in the current year that have not yet accumulated many citations may not have been fully represented. Despite these limitations, the literature analyzed in this study still provides a systematic overview of the latest research progress on the meningeal lymphatic system in the field of NDs, highlighting major research directions and future trends.

## 5 Conclusion

This analytical framework provides a powerful tool for the systematic study of the knowledge diffusion pathways and cutting-edge advancements in the glymphatic system within NDs. Over the past thirteen years, research on the glymphatic system in NDs has shown a rapid developmental trend globally. The significant increase in the number of annual publications fully demonstrates that the importance of research in this field is becoming increasingly prominent. In this field, the most influential scholar is Nedergaard Maiken, the most representative journals are the Frontiers series, and the University of Rochester in the United States is the most influential institution. Current research hotspots are mainly focused on the metabolic clearance mechanisms mediated by AQP4 polarization, as well as the regulatory roles of sleep, aging, and inflammation. The primary disease models involved include AD and PD. The frontier research directions point towards non-invasive imaging technologies such as multimodal MRI (Zhou, 2016) and AI-driven intervention paradigms as well as multiomic confirmations (Zhou, 2021). Looking ahead, research on the glymphatic system in NDs should focus on promoting multicenter, large-sample international collaborative studies. This study examines the developmental trends of research on this topic from the perspective of bibliometrics and provides a comprehensive summary of the research hotspots in this field. Moreover, these findings also offer valuable references for researchers to select appropriate publication channels and establish scientific research partnerships.
